# A genome-wide association study of body mass index across early life and childhood

**DOI:** 10.1093/ije/dyv077

**Published:** 2015-05-07

**Authors:** Nicole M Warrington, Laura D Howe, Lavinia Paternoster, Marika Kaakinen, Sauli Herrala, Ville Huikari, Yan Yan Wu, John P Kemp, Nicholas J Timpson, Beate St Pourcain, George Davey Smith, Kate Tilling, Marjo-Riitta Jarvelin, Craig E Pennell, David M Evans, Debbie A Lawlor, Laurent Briollais, Lyle J Palmer

**Affiliations:** ^1^School of Women’s and Infants’ Health, University of Western Australia, Perth, WA, Australia, ^2^University of Queensland Diamantina Institute, Translational Research Institute, Brisbane, QLD, Australia, ^3^MRC Integrative Epidemiology Unit, University of Bristol, Bristol, UK, ^4^School of Social and Community Medicine, University of Bristol, Bristol, UK, ^5^Biocenter Oulu, and ^6^Institute of Health Sciences, University of Oulu, Oulu, Finland, ^7^Department of Genomics of Common Disease, School of Public Health, Imperial College London, London, UK, ^8^Lunenfeld-Tanenbaum Research Institute, Mount Sinai Hospital, Toronto, ON, Canada, ^9^Department of Children and Young People and Families, National Institute for Health and Welfare, Oulu, Finland, ^10^Department of Epidemiology and Biostatistics, MRC-HPA Centre for Environment and Health, School of Public Health, Imperial College London, London, UK, ^11^Unit of Primary Care, Oulu University Hospital, Oulu, Finland and ^12^The Joanna Briggs Institute, The Robinson Research Institute, and School of Translational Health Science, University of Adelaide, Adelaide, SA, Australia

**Keywords:** Body mass index, genome-wide association study, trajectory, childhood, ALSPAC, Raine

## Abstract

**Background:** Several studies have investigated the effect of known adult body mass index (BMI) associated single nucleotide polymorphisms (SNPs) on BMI in childhood. There has been no genome-wide association study (GWAS) of BMI trajectories over childhood.

**Methods:** We conducted a GWAS meta-analysis of BMI trajectories from 1 to 17 years of age in 9377 children (77 967 measurements) from the Avon Longitudinal Study of Parents and Children (ALSPAC) and the Western Australian Pregnancy Cohort (Raine) Study. Genome-wide significant loci were examined in a further 3918 individuals (48 530 measurements) from Northern Finland. Linear mixed effects models with smoothing splines were used in each cohort for longitudinal modelling of BMI.

**Results:** A novel SNP, downstream from the *FAM120AOS* gene on chromosome 9, was detected in the meta-analysis of ALSPAC and Raine. This association was driven by a difference in BMI at 8 years (T allele of rs944990 increased BMI; P_SNP_ = 1.52 × 10^−8^), with a modest association with change in BMI over time (P_Wald(Change)_ = 0.006). Three known adult BMI-associated loci (*FTO*, *MC4R* and *ADCY3*) and one childhood obesity locus (*OLFM4*) reached genome-wide significance (P_Wald_ < 1.13 × 10^−8^) with BMI at 8 years and/or change over time.

**Conclusions:** This GWAS of BMI trajectories over childhood identified a novel locus that warrants further investigation. We also observed genome-wide significance with previously established obesity loci, making the novel observation that these loci affected both the level and the rate of change in BMI. We have demonstrated that the use of repeated measures data can increase power to allow detection of genetic loci with smaller sample sizes.

**Key Messages**

- We performed a genome-wide association study of body mass index trajectories over childhood and adolescence using repeated measures data from two large cohort studies.
- A novel association between body mass index at 8 years of age and single nucleotide polymorphisms near the *FAM120AOS* gene on chromosome 9 was identified. The top SNP in this region begins to have an observable effect on BMI around 2 years of age, and the effect appears to be driven by changes in weight rather than height. This association warrants further investigation.
- Novel associations between several known adult body mass index-associated loci and body mass index at 8 years of age and/or change in body mass index over childhood were observed. In addition, a known childhood obesity-associated locus was associated with body mass index at 8 years of age.
- Through the use of repeated measures data, genetic associations can be detected with smaller sample sizes.
- Our results highlight that the genetic determinants of susceptibility to obesity in adulthood begin acting in early life and develop over the life course.

## Introduction

Obesity, defined by high body mass index (BMI), is a complex condition associated with an increased risk of many chronic diseases.^1^ Evidence suggests that the adverse health consequences of obesity begin in early life.^2–4^ BMI in adults primarily reflects weight independent of height; however, changes in BMI throughout childhood are influenced by changes in both height and weight.

Choh *et al*.^5^ estimated that the narrow-sense heritability of BMI over infancy, childhood and adolescence ranges from 47% to 76%, with similar estimates reported in adults.^6^^,^^7^ Genome-wide association studies (GWAS) have identified and replicated 34 adult BMI-associated loci (*P*-values< 5 × 10^−8^ from discovery and replication meta-analysis)^8–10^ that explain approximately 1.5% of the variance in adult BMI. Several studies have investigated the association between these adult BMI SNPs and childhood BMI, including BMI trajectories.^11–15^ These publications have shown that SNPs associated with adult BMI are not associated with birthweight or length, but have effects that begin from early infancy and strengthen throughout childhood and adolescence. The effects of some variants appear to change over time, for example the A allele at the *FTO* rs9939609 SNP is associated with a lower BMI in infancy but a higher BMI from 5.5 years onwards.^16^ In contrast, the *ADCY3* rs11676272 SNP has been shown to have consistent effects across ages.^17^ A GWAS investigating childhood obesity (i.e. BMI > 95th percentile) in populations of European descent^18^ identified two novel loci, one near the olfactomedin 4 (*OLFM4*) gene and the other in the homeobox B5 (*HOXB5*) gene. The results of these studies suggest that the expression of genetic variants may change across the life course.

In a recent review of obesity genetics, Day and Loos highlight the importance of conducting GWAS in children and adolescents to identify loci that may have effects early in life rather than adulthood.^19^ Therefore, the aim of the current study was to investigate the genetic basis of BMI and BMI trajectories across childhood and adolescence.

## Methods

### Participants

#### 

##### ALSPAC

The Avon Longitudinal Study of Parents and Children (ALSPAC) is a prospective cohort. Details on the BMI measurements are provided in Supplementary material (available as Supplementary data at *IJE* online) and the full study methodology is published elsewhere^20^ [www.bristol.ac.uk/alspac]. The study website contains details of all the data that are available through a fully searchable data dictionary [http://www.bris.ac.uk/alspac/researchers/data-access/data-dictionary]. A subset of 7916 individuals (69 297 observations) were included in this study based on the following criteria: ≥1 parent of European descent, live singleton birth, unrelated to anyone in the sample, no major congenital anomalies, genotype data, and ≥1 measure of BMI between ages 1 and 17 years. Ethical approval for the study was obtained from the ALSPAC Law and Ethics Committee and the local research ethics committees.

##### Raine

The Western Australian Pregnancy Cohort (Raine) Study^21–23^ is a prospective pregnancy cohort [http://www.rainestudy.org.au/] (specific methods in Supplementary material, available as Supplementary data at *IJE* online). A subset of 1461 individuals (8670 observations) was included in this study, using the same criteria as in the ALSPAC cohort. The study was conducted with institutional ethics approval from the King Edward Memorial Hospital and Princess Margaret Hospital for Children human research ethics committees.

### Genotyping

Imputed genotypic data used in both cohorts has been described previously.^24^^,^^25^ Briefly, genotype data in both cohorts were cleaned using standard thresholds (HWE *P* > 5.7 × 10^−7^, call rate >95% and minor allele frequency >1%). Imputation for chromosomes 1 to 22 was performed with the MACH software^26^ using the CEU samples from HapMap Phase 2 (Build 36, release 22) as a reference panel.

### Within-study GWAS analysis and quality control

BMI was skewed, so a natural log transformation was applied before analysis. A semi-parametric linear mixed model was fitted to the BMI measures with mean centred age (centred at age 8); smoothing splines, with knot points at two, eight and 12 years and a cubic slope for each spline, were used to produce a smooth growth curve estimate.^27^ A continuous autoregressive of order 1 correlation structure was assumed. Full details of the statistical methodology are in Supplementary material, available as Supplementary data at *IJE* online.

The fixed effects in the ALSPAC model included a binary indicator of measurement source (questionnaire vs clinic or health visitor measurement) to allow for differential measurement error. The fixed effects in the Raine model included the first five principal components for population stratification, calculated in EIGENSTRAT;^28^ no adjustment for population stratification was made in ALSPAC, as previous analyses have shown that there is no obvious stratification.

A recent GWAS meta-analysis of adults from the Genetic Investigation of ANthropometric Traits (GIANT) consortium has shown that there were no genome-wide significant gender difference in SNP-BMI associations.^29^ Therefore, the sexes were combined with the inclusion of a main sex effect and an interaction between sex and the spline function for age. This allowed the average BMI trajectory to differ between males and females, but with genetic effects assumed to be consistent across sexes.

The semi-parametric linear mixed model was fitted for each individual SNP. Genetic differences in the trajectories were estimated by including both a main effect for the imputed dosage and an interaction between the spline function for age and the imputed dosage for each genetic variant (i.e. an additive genetic model). We have previously shown that the type 1 error of the genetic effect over time in linear mixed effects models may be inflated if the function involving age terms in the fixed and random effects differs.^30^ Therefore, a robust standard error and a corresponding *P*-value was calculated for each fixed effect parameter (details in Supplementary material, available as Supplementary data at *IJE* online).

To understand the relationship between each SNP and BMI trajectory, three test statistics were calculated (details in Supplementary material, available as Supplementary data at *IJE* online).

- Global test (Wald test): to test the association of SNPs with BMI from 1–17 years of age (denoted as Wald). This tests the association between an SNP and any change in BMI.
- SNP by age interaction (Wald test): to test the association of SNPs with change in BMI trajectory between 1–17 years of age [denoted as Wald(Change)]. This tests the association between an SNP and any change in the shape of the BMI curve.
- SNP effect at age 8: to test the association of SNPs with childhood BMI at age 8 years (referred to as ‘BMI intercept at 8 years’ and denoted as SNP). This represents the test for a shift up or down of the whole trajectory due to a SNP. The effect is estimated at 8 years as the age data are mean centred before entering the semi-parametric linear mixed model.

All analyses were conducted in *R* [version 3.0.2 (2013-09-25)]^31^ using the nlme package. SNPs were excluded if their MAF was <5% or imputation quality (R^2^ from MACH) was <0.3.

### Meta-analysis

The meta-analysis for the SNP effect at age 8 years was conducted in Metal^32^ [http://www.sph.umich.edu/csg/abecasis/metal/], using inverse variance weighting. The analysis in Metal included an adjustment for genomic control in both cohorts, and a test of heterogeneity of the effect sizes was carried out.

Stouffer’s method for combining *P*-values was used for the global Wald test and the Wald test for the SNP by age interaction.^33^ Genomic inflation λ was estimated by dividing the chi-square statistics, with 7 degrees of freedom for the global Wald test and 6 degrees of freedom for the SNP by age interaction Wald test, by the median of the central ${\text{χ}}_{7}^{2}$ and ${\text{χ}}_{6}^{2}$ distributions, respectively.^34^ *P*-values from ALSPAC and Raine were adjusted for the estimated λ values and weighted by the square root of the respective samples sizes in the meta-analysis.

### Follow-up

The Northern Finland Birth Cohort of 1966 (NFBC1966) was used to replicate regions that reached genome-wide significance (*P* < 5 × 10^−8^) on any one of the three tests conducted. We investigated the SNP with the lowest *P*-value in each region and any potentially functional SNPs that were in high linkage disequilibrium (LD) with that SNP; LD was determined from SNAP^35^ [http://www.broadinstitute.org/mpg/snap/ldsearchpw.php], and functionality from snpinfo^36^ [http://snpinfo.niehs.nih.gov/snpinfo/snpfunc.htm]. NFBC1966 is a prospective birth cohort from the region covering the Provinces of Lapland and Oulu, Finland^37^ [http://kelo.oulu.fi/NFBC/] (specific methods in Supplementary material, available as Supplementary data at *IJE* online). A subset of 3918 individuals with growth data (48 530 observations) was included in this study, using the same criteria as in the discovery cohorts. The study was approved by ethics committees in Oulu (Finland) and Oxford (UK) universities in accordance with the Declaration of Helsinki.

### Characterization of significant loci

For loci reaching genome-wide significance, we plotted the trajectory for each of the genotypes and estimated the earliest age at which the effect of the SNP could be detected. For novel SNPs, we also investigated their association with height and weight trajectories using a spline model similar to that used for BMI (see ref. 11 for description of methods used). These additional analyses were conducted in the ALSPAC study only, being the largest discovery cohort.

## Results

Each cohort has a similar proportion of males and females; NFBC1966 has a lower average weight, and consequently BMI, than the two discovery cohorts from approximately 6 years of age, whereas their average height is similar (Table 1, and Supplementary Figure 1 available as Supplementary data at *IJE* online). The meta-analysis of approximately 2.2 million SNPs indicated that the lowest observed *P*-values for each of the three test statistics deviated from the expected null distribution (Supplementary Figure 2, available as Supplementary data at *IJE* online), whereas any systematic inflation of the test statistics was negligible (λ_SNP_ = 1.03, λ_Wald_ = 1.00, λ_Wald(Change)_ = 1.00). There was little heterogeneity between the discovery cohorts (Supplementary Figure 3, available as Supplementary data at *IJE* online).

**Table 1.** Characteristics of individuals in the cohorts involved in the discovery meta-analysis and replication

Sex (% male)	Age stratum	Discovery cohorts				Replication cohort	
		ALSPAC (*N* = 7,916; n = 69,297)		Raine (*N* = 1,461; *n* = 8,670)		NFBC1966 (*N* = 3,918; *n* = 48,530)	
		51.27		51.54		49.74	
		***N***	**Mean (SD)**	***N***	**Mean (SD)**	***N*****^a^**	**Mean (SD)**
BMI (kg/m^2^)	1–1.49	2372	17.42 (1.51)	1326	17.11 (1.39)	3304	17.66 (1.57)
	1.5–2.49	5490	16.82 (1.49)	389	16.00 (1.25)	2769	16.81 (1.43)
	2.5–3.49	1679	16.48 (1.40)	957	16.14 (1.24)	2252	16.13 (1.29)
	3.5–4.49	5949	16.25 (1.39)	20	15.92 (1.41)	2194	15.76 (1.32)
	4.5–5.49	1602	16.02 (1.70)	3	15.94 (1.43)	1967	15.47 (1.31)
	5.5–6.49	3322	15.71 (1.87)	1273	15.85 (1.70)	1870	15.44 (1.54)
	6.5–7.49	2728	16.10 (1.98)	43	16.64 (2.85)	3040	15.56 (1.61)
	7.5–8.49	3588	16.30 (2.01)	1045	16.89 (2.52)	2629	15.89 (1.76)
	8.5–9.49	4376	17.15 (2.41)	205	16.95 (2.57)	1965	16.32 (2.00)
	9.5–10.49	5516	17.67 (2.81)	304	18.95 (3.43)	2056	16.88 (2.24)
	10.5–11.49	4710	18.25 (3.10)	933	18.66 (3.41)	2619	17.32 (2.42)
	11.5–12.49	5116	19.04 (3.35)	4	16.78 (2.65)	2911	17.80 (2.53)
	12.5–13.49	5268	19.64 (3.35)	9	21.11 (3.76)	2175	18.58 (2.67)
	13.5–14.49	4603	20.30 (3.44)	1201	21.46 (4.17)	2383	19.25 (2.71)
	14.5–15.49	2342	21.28 (3.48)	24	21.66 (4.23)	2387	19.77 (2.66)
	15.5–16.49	1659	21.39 (3.51)	2	20.14 (3.26)	1191	20.47 (2.88)
	16.5+	91	22.46 (3.39)	931	23.02 (4.37)	970	20.83 (2.66)
Weight (kg)	1–1.49	2372	10.56 (1.31)	1326	10.33 (1.22)	3304	10.52 (1.25)
	1.5–2.49	5490	12.01 (1.49)	389	12.98 (1.49)	2769	12.29 (1.46)
	2.5–3.49	1679	14.72 (1.89)	957	15.03 (1.81)	2252	14.34 (1.69)
	3.5–4.49	5949	16.51 (2.03)	20	15.34 (1.94)	2194	16.27 (2.01)
	4.5–5.49	1602	19.42 (2.83)	3	21.97 (1.90)	1967	18.17 (2.34)
	5.5–6.49	3322	21.32 (3.37)	1273	21.45 (3.27)	1870	20.39 (3.01)
	6.5–7.49	2728	24.98 (4.29)	43	26.00 (5.89)	3040	22.62 (3.46)
	7.5–8.49	3588	26.42 (4.68)	1045	28.26 (5.62)	2629	25.04 (4.10)
	8.5–9.49	4376	30.33 (5.79)	205	29.75 (5.85)	1965	28.37 (4.88)
	9.5–10.49	5516	34.71 (7.30)	304	39.24 (9.06)	2056	31.88 (5.90)
	10.5–11.49	4710	38.26 (8.46)	933	39.07 (9.07)	2619	35.32 (6.77)
	11.5–12.49	5116	43.60 (9.91)	4	35.85 (6.32)	2911	39.05 (7.78)
	12.5–13.49	5268	49.41 (10.58)	9	55.34 (11.15)	2175	44.82 (8.88)
	13.5–14.49	4603	54.43 (11.11)	1201	58.47 (13.17)	2383	49.77 (9.14)
	14.5–15.49	2342	61.05 (11.85)	24	61.06 (12.74)	2387	53.71 (9.22)
	15.5–16.49	1659	61.75 (11.64)	2	57.63 (13.40)	1191	58.02 (9.80)
	16.5+	91	64.64 (11.74)	931	68.79 (14.62)	970	59.94 (9.35)
Height (m)	1–1.49	2372	0.78 (0.04)	1326	0.78 (0.03)	3304	0.77 (0.03)
	1.5–2.49	5490	0.84 (0.04)	389	0.90 (0.04)	2769	0.85 (0.04)
	2.5–3.49	1679	0.94 (0.04)	957	0.96 (0.04)	2252	0.94 (0.04)
	3.5–4.49	5949	1.01 (0.04)	20	0.98 (0.04)	2194	1.02 (0.04)
	4.5–5.49	1602	1.10 (0.05)	3	1.18 (0.08)	1967	1.08 (0.05)
	5.5–6.49	3322	1.16 (0.06)	1273	1.16 (0.05)	1870	1.15 (0.05)
	6.5–7.49	2728	1.24 (0.06)	43	1.25 (0.06)	3040	1.20 (0.05)
	7.5–8.49	3588	1.27 (0.06)	1045	1.29 (0.06)	2629	1.25 (0.06)
	8.5–9.49	4376	1.33 (0.06)	205	1.32 (0.05)	1965	1.32 (0.06)
	9.5–10.49	5516	1.40 (0.06)	304	1.43 (0.06)	2056	1.37 (0.06)
	10.5–11.49	4710	1.44 (0.07)	933	1.44 (0.07)	2619	1.42 (0.07)
	11.5–12.49	5116	1.51 (0.07)	4	1.46 (0.05)	2911	1.48 (0.07)
	12.5–13.49	5268	1.58 (0.08)	9	1.62 (0.06)	2175	1.55 (0.08)
	13.5–14.49	4603	1.63 (0.08)	1201	1.65 (0.08)	2383	1.60 (0.08)
	14.5–15.49	2342	1.69 (0.08)	24	1.68 (0.06)	2387	1.65 (0.08)
	15.5–16.49	1659	1.70 (0.08)	2	1.69 (0.06)	1191	1.68 (0.08)
	16.5+	91	1.69 (0.08)	931	1.73 (0.10)	970	1.70 (0.08)

^a^Due to the data structure in NFBC1966, some individuals had multiple measures within a 1-year period. This column is the number of unique individuals with measures in the age bracket.

Five regions reached genome-wide significance at 5 × 10^−8^ (Table 2, and Supplementary Table 1 available as Supplementary data at *IJE* online); three of these loci have previously been identified through GWAS of adult BMI (*FTO,*^38^ *MC4R*^39^ and *ADCY3* ^8^) and one locus has previously been shown to be associated with paediatric obesity (*OLFM4.*^18^). Manhattan plots for each of the three tests are in Supplementary Figures 4–6, (available as Supplementary data at *IJE* online).

**Table 2.** Genome wide significant (*P* < 5 × 10^−8^) loci for the three tests investigated from the meta-analysis, showing the most significant SNP from each locus

SNP	Nearest gene	Chr.	Position^a^ (bp)	Alleles		Effect allele frequency^b^		ALSPAC *P*-value	Raine *P*-value	Meta-analysis *P*-value	Het I^2^ (*P*-value)	NFBC1966 *P-*value	Discovery + replication *P*-value^c^
				Effect	Other								
rs11676272	*ADCY3*	2	25003800	A	G	0.514	SNP	1.08 × 10^−7^	0.029	8.94 × 10^−9^	0 (0.999)	2.40 × 10^−4^	1.18 × 10^−11^
							Wald	2.94 × 10^−11^	0.110	1.15 × 10^−9^		0.007	1.12 × 10^−10^
							Wald (interaction)	0.080	0.645	0.199		0.102	0.081
rs944990	*FAM120A/FAM120AOS*	9	95230825	C	T	0.723	SNP	4.48 × 10^−7^	0.009	1.52 × 10^−8^	0 (0.645)	0.109	5.98 × 10^−8^
							Wald	2.93 × 10^−5^	0.201	1.51 × 10^−4^		0.108	1.05 × 10^−4^
							Wald (interaction)	0.003	0.210	0.006		0.134	0.003
rs12429545	*OLFM4*	13	53000207	A	G	0.128	SNP	1.98 × 10^−8^	0.244	1.90 × 10^−8^	25 (0.248)	0.019	9.68 × 10^−9^
							Wald	6.94 × 10^−10^	0.090	1.13 × 10^−8^		0.031	5.58 × 10^−9^
							Wald (interaction)	1.28 × 10^−5^	0.069	3.08 × 10^−5^		0.051	1.06 × 10^−5^
rs1558902	*FTO*	16	52361075	A	T	0.402	SNP	7.62 × 10^−8^	1.75 × 10^−4^	1.54 × 10^−10^	52 (0.149)	2.94 × 10^−7^	2.22 × 20^−16^
							Wald	4.81 × 10^−24^	0.010	1.48 × 10^−21^		4.48 × 10^−7^	1.24 × 10^−26^
							Wald (interaction)	1.02 × 10^−24^	0.006	4.99 × 10^−22^		1.23 × 10^−6^	1.45 × 10^−26^
rs571312	*MC4R*	18	55990749	A	C	0.234	SNP	3.54 × 10^−8^	0.361	4.94 × 10^−8^	35 (0.215)	0.280	5.23 × 10^−7^
							Wald	5.10 × 10^−11^	0.085	1.27 × 10^−9^		0.080	3.95 × 10^−9^
							Wald (interaction)	1.75 × 10^−10^	0.059	2.81 × 10^−9^		0.064	5.26 × 10^−9^

Chr, chromosome; bp, basepair.

^a^Positions according to Build 36.

^b^Average effect allele frequency reported in the Metal meta-analysis of ALSPAC and Raine.

^c^*P*-value from the meta-analysis of ALSPAC, Raine and NFBC66.

**Figure 1.**
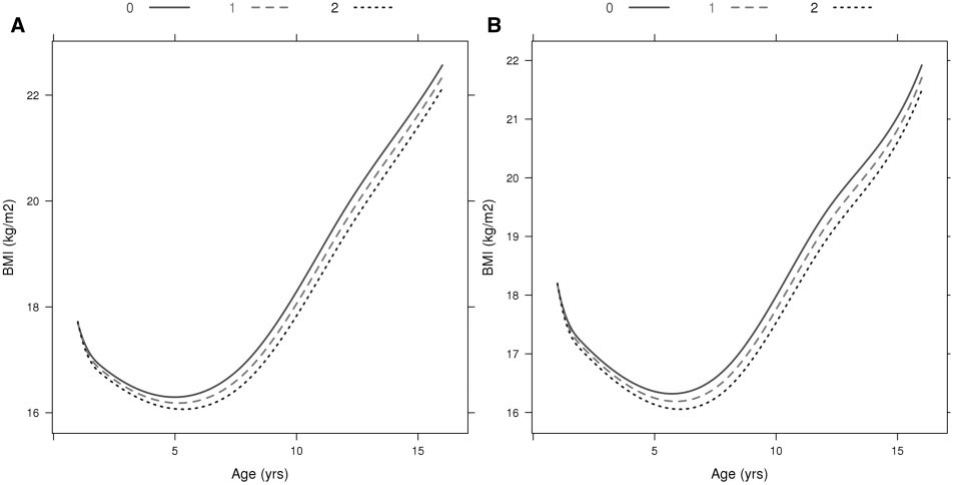
Population average trajectories for females (A) and males (B) from the ALSPAC cohort with 0, 1 or 2 copies of the C allele at the *FAM120AOS*, rs944990, locus.

**Figure 2.**
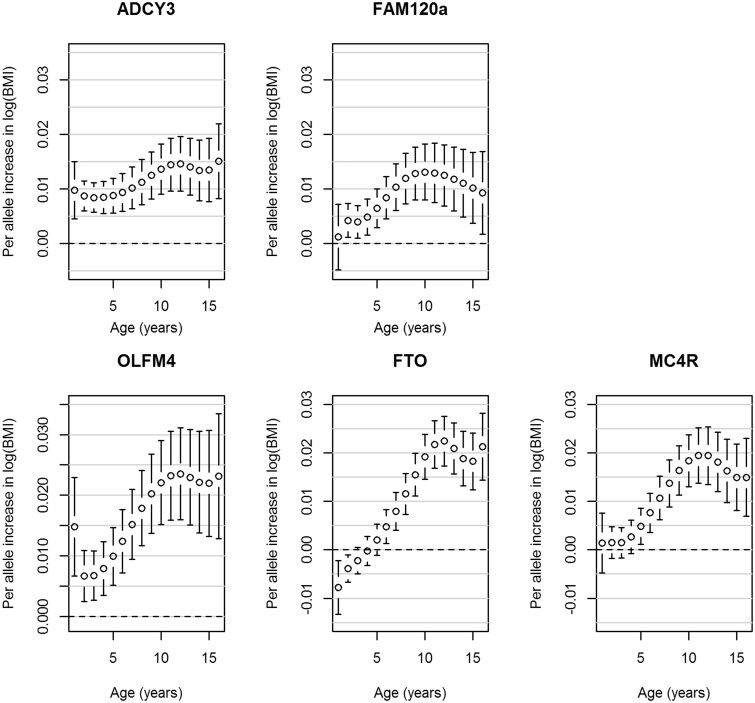
Associations from the ALSPAC cohort between the genome-wide significant SNPs and BMI from age one to 16 years. Error bars represent the regression coefficient of BMI on the natural log scale and 95% confidence intervals derived from the longitudinal additive genetic models. The SNPs are aligned to the minor allele.

**Figure 3.**
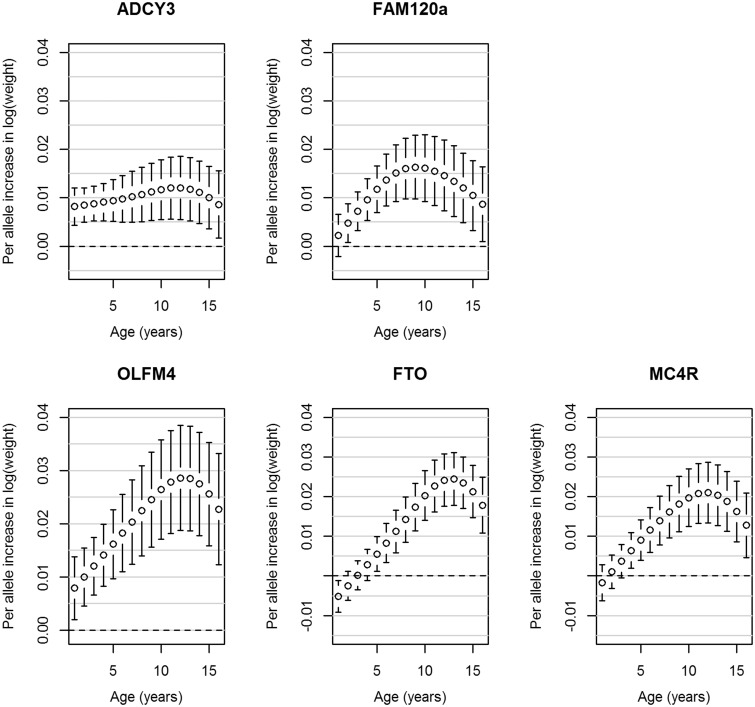
Associations from the ALSPAC cohort between the genome-wide significant SNPs and weight from age one to 16 years. Error bars represent the regression coefficient of weight on the natural log scale and 95% confidence intervals derived from the longitudinal additive genetic models. The SNPs are aligned to the minor allele.

**Figure 4.**
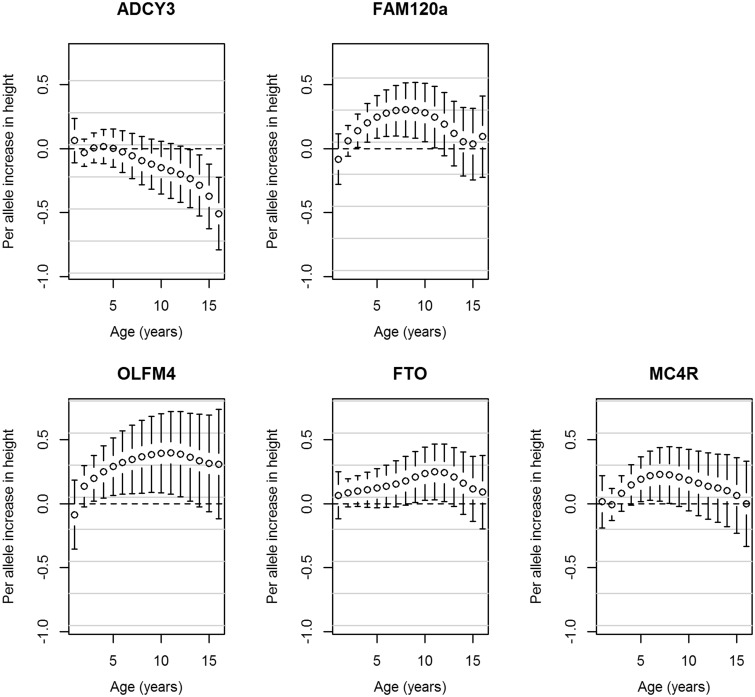
Associations from the ALSPAC cohort between the genome-wide significant SNPs and height from age one to 16 years. Error bars represent the regression coefficient of height and 95% confidence intervals derived from the longitudinal additive genetic models. The SNPs are aligned to the minor allele.

A novel genome-wide significant locus was found downstream from *FAM120AOS*, which has not previously been reported to be associated with any adiposity-related traits. The most statistically significant SNP, rs944990, was associated with BMI intercept at 8 years (T allele: β_SNP_ = 0.012, P_SNP_ = 1.52 × 10^−8^), and showed modest evidence of association with change in BMI (P_Wald(Change)_ = 0.006; Supplementary Figure 9, available as Supplementary data at *IJE* online). A consistent direction of effect was seen in NFBC1966 for rs944990; however, *P* > 0.05 for all three tests (Table 2). Figure 1 shows the BMI trajectories in the ALSPAC cohort from 1 to 17 years of age in males and females for individuals who have zero, one or two BMI decreasing alleles (major allele) at the rs944990 locus. A male homozygous for the T allele would have an average BMI of 18.16 kg/m^2^ at 1 year of age, which would increase to an average of 21.88 kg/m^2^ by age 16. In contrast, a male homozygous for the C allele would on average have a BMI of 18.12 kg/m^2^ at 1 year of age and by age 16 it would be 21.48 kg/m^2^. In ALSPAC, the effect is detectable from 2 years of age (Figure 2). Consistent results were observed after including additional fixed effects in the model to account for change in height (Supplementary Table 2, available as Supplementary data at *IJE* online). rs944990 showed a stronger association with weight than height (Supplementary Table 2) and had the strongest influence on both weight and height over the pre-pubertal years (7–10 years; Figures 3 and 4). We investigated the association between energy intake and rs944990 from 3 to 14 years of age in the ALSPAC cohort (Supplementary Table 3, available as Supplementary data at *IJE* online). Assuming an additive effect of the SNP, there was a marginal association at 39 months of age (β = 14.28 kCal per T allele, *P* < 0.001) and only a slight increase in energy intake was observed around the peak of the BMI association (10 years of age; β = 12.80 kCal per T allele, *P* = 0.03).

The most statistically significant SNP at this novel *FAM120AOS* locus in our meta-analysis, rs944990, is in LD (r^2 ^= 0.72, D’ = 0.96^35^) with a non-synonymous variant that may disrupt splicing activity^36^, rs10821128. In the meta-analysis, the C allele of rs10821128 was associated with an increase in BMI intercept at age 8 years (β_SNP_ = 0.011, *P*_SNP_ = 5.29 × 10^−8^) and showed weak evidence for an increased change in BMI (*P*_Wald_ = 1.14 × 10^−4^; *P*_Wald(Change)_ = 0.014). Given the potential functionality of this SNP, we analysed rs10821128 in the NFBC1966 cohort. Consistent with the results from the meta-analysis, the C allele at rs10821128 was associated with BMI intercept at 8 years of age in NFBC1966 (β_SNP_ = 0.005, *P*_SNP_ = 0.041) and the global Wald test (*P*_Wald_ = 0.041), but not with change in BMI (*P*_Wald(Change)_ = 0.139). Neither this SNP nor rs944990 was associated with mean BMI (rs944990: *P* = 0.887; rs10821128: *P* = 0.931) in the GIANT consortium meta-analysis^8^ but showed weak evidence of association with phenotypic variability of BMI^40^ (rs944990: *P* = 0.063; rs10821128: *P* = 0.092). We used publicly available results from GWAS in various consortia to investigate whether rs944990 or rs10821128 was associated with any other growth-related endpoint (including BMI, height, waist to hip ratio, obesity, glucose, insulin, homeostatic model assessment–insulin resistance (HOMA-IR) and type 2 diabetes in adults, age of menarche, pubertal growth and obesity in children and birthweight, birth length and head circumference at birth). Both rs944990 (*P* = 4.0 × 10^−4^) and rs10821128 (*P* = 5.9x10^−4^) were associated with age of menarche, and rs10821128 was marginally associated with total pubertal growth (*P* = 0.044), consistent with the observation that rs944990 influences pre-pubertal weight and height growth (Figures 3 and 4). Supplementary Table 4 (available as Supplementary data at *IJE* online) presents the association results for other endpoints.

SNPs in *FTO* (Supplementary Figures 12 and 13, available as Supplementary data at *IJE* online) and *MC4R* (Supplementary Figures 14 and 15, available as Supplementary data at *IJE* online) reached genome-wide significance for all three tests, indicating that these loci influence both BMI intercept at 8 years and change in BMI trajectory over childhood (*FTO* rs1558902: β_SNP_ = 0.013, *P*_SNP_ = 1.54 × 10^−10^; *P*_Wald_ = 1.48 × 10^−21^; *P*_Wald(Change)_ = 4.99 × 10^−22^; *MC4R* rs571312: β_SNP_ = 0.013, *P*_SNP_ = 4.94 × 10^−8^; *P*_Wald_ = 1.27 × 10^−9^; *P*_Wald(Change)_ = 2.81 × 10^−9^). The A allele at rs1558902 in *FTO* was associated with a decrease in BMI until 2 years of age, but an increase in BMI from 6 years of age (Figure 2 and Supplementary Figure 13).

The *OLFM4* rs12429545 SNP reached genome-wide significance for the SNP effect at age 8 (β_SNP_ = 0.016, *P*_SNP_ = 1.90 × 10^−8^) and global Wald tests (*P*_Wald_ = 1.13 × 10^−8^) and showed weak evidence for association with change in BMI over time (Supplementary Figures 10 and 11, available as Supplementary data at *IJE* online).

A missense variant located in *ADCY3* reached genome-wide significance for the SNP effect at age 8 (rs11676272: β_SNP_ = −0.011, *P*_SNP_ = 8.94 × 10^−9^) and global Wald tests (*P*_Wald_ = 1.15 × 10^−9^) but not for the SNP by age interaction terms (*P*_Wald(Change)_ = 0.199; Table 2).

**Figure 5.**
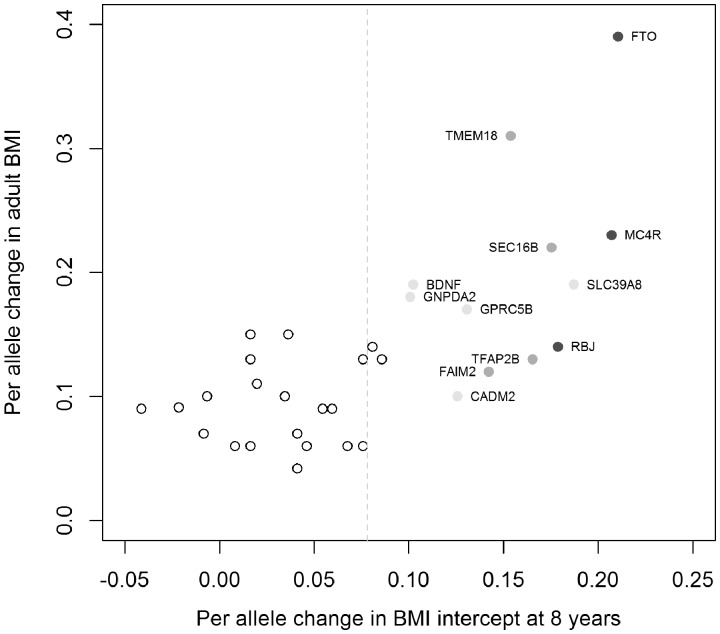
Association results of SNP effect at age 8 years for known adult BMI-associated loci. The y-axis displays the effect size from published meta-analyses^8–10^ and the x-axis displays the effect size from the meta-analysis presented in this paper of the BMI intercept at age 8. Effect sizes are aligned to the adult BMI-increasing allele. Colour indicates association *P*-values from the longitudinal model over childhood: dark grey = *P* < 5 × 10^−8^, medium grey = 5 × 10^−8 ^≤ *P* < 0.001, light grey = 0.001 ≤ *P* < 0.01, white = *P* ≥ 0.01. The dotted grey line indicates the mean effect size for the 33 SNPs in the longitudinal model parameters. The effect sizes from the meta-analysis were transformed from the log scale^49^ using an intercept of 2.80 and standard deviation of 0.057, for comparison with the adult effect sizes.

To investigate whether known adult BMI-associated loci influenced BMI trajectory through childhood and adolescence, we investigated the relationship between the 33 top adult BMI-associated SNPs and the SNP effect at age 8 in our meta-analysis. One SNP from the adult BMI GWAS, rs11847697, was not available in our study as it had a minor allele frequency less than 5%. We observed 15 loci with directionally consistent associations at nominal significance (*P* < 0.05), 12 of which reached *P* < 0.01. Figure 5 indicates that the majority of the loci show the same direction of effect for both the SNP effect at age 8 and the adult BMI meta-analyses. Results from the meta-analysis of the three tests for the 33 adult BMI-associated SNPs are presented in Supplementary Figure 16 and Supplementary Table 5 (available as Supplementary data at *IJE* online). We also looked up the *HOXB5* and *OLFM4* SNPs, previously associated with childhood obesity.^18^ The obesity risk T allele at rs9299 in *HOXB5* increased BMI intercept at 8 years (β_SNP_ = 0.004, *P*_SNP_ = 0.068), but was not associated with change in BMI over time (*P*_Wald_ = 0.591; *P*_Wald(Change)_ = 0.728). The obesity risk A allele at rs9568856 near *OLFM4* was associated with increased BMI intercept at 8 years (β_SNP_ = 0.014, *P*_SNP_ = 1.68 × 10^−6^) and increased change in BMI (*P*_Wald_ = 3.24 × 10^−6^; *P*_Wald(Change)_ = 0.002).

## Discussion

Previous studies have shown that some genetic variants for BMI have varying effects across the life course, suggesting potential age-specific gene expression.^14–17^ We conducted a genome-wide meta-analysis of BMI trajectories from 1 to 17 years of age and identified a novel association with rs944990, downstream from *FAM120AOS* on chromosome 9. This SNP was associated with BMI intercept at 8 years, an effect that was independent of height. The effect size for the T allele, 0.20 kg/m^2^ in ALSPAC, is comparable to the effect sizes reported for adult BMI-associated SNPs.^8^ In addition, rs944990 showed a stronger association with adiposity than skeletal growth and had the greatest influence on pre-pubertal growth. This association did not replicate in the NFBC1966 cohort; however, a potentially functional non-synonymous SNP in LD was associated with BMI intercept in NFBC1966. The lack of convincing evidence for association in NFBC1966 could be due to the observed difference in BMI trajectories to the ALSPAC and Raine cohorts (as seen in Supplementary Figure 1); with fewer individuals in the right-hand tail of the BMI distribution, it is more difficult to detect an association. This difference in trajectories could be due to several causes, including different genetic profiles or generational effects.

Little is known about the function of *FAM120AOS.* However, there are two genes in the region surrounding *FAM120AOS* that have shown evidence to support a role for this region in growth. An SNP in the first of those genes, *NINJ1*, has been shown to be associated with severe obesity in children.^41^ The second gene, *PHF2*, has been shown to influence bone development in newborn mice^42^ and adipocyte differentiation.^43^ In addition to these two plausible candidate genes in the region surrounding *FAM120AOS*, we identified that the T allele at rs944990 was associated with increased BMI, increased change in BMI and earlier age of menarche. The direction of effect in this study is consistent with the observed phenotypic correlation,^44^^,^^45^ where girls who reach menarche earlier tend to have a higher BMI in later life than girls who reach menarche later. The effect seen by rs944990 is similar to that reported for the *LIN28B* locus, whereby it influences BMI and weight from adolescence to early/mid adulthood, and is associated with age of menarche.^46^ Therefore, we believe this region of chromosome 9 warrants further investigation.

Four loci previously associated with either adult BMI (*FTO*,^38^ *MC4R*^39^ and *ADCY3*^8^) or childhood obesity (*OLFM4*^18^) reached genome-wide significance for BMI level and/or slope. These loci were detected with a smaller sample size than the original studies (>20 000 subjects in ref. 18 and 249 796 subjects in ref. 8), consistent with the increased statistical power gained through using repeated measures.^47^ A small increase in BMI may not necessarily lead to obesity; however, our results demonstrate for the first time that the obesity risk allele of the *OLFM4* rs9568856 SNP^18^ is associated with increased BMI at 8 years and increased change in BMI over childhood, rather than having an effect on obesity only. This SNP, along with the top SNPs from the *FTO* and *MC4R* loci, had an effect at baseline as well as showing an increasing effect over childhood. In contrast, the top SNP at *ADCY3* was associated with baseline and its effect over time was relatively constant. These results are consistent with previous studies on adult BMI loci.^16^^,^^17^^,^^39^ Furthermore, we detected a decrease in BMI in infancy for the *FTO* SNP, rs1558902, followed by an increase from early childhood: a result which is consistent with our previous work.^16^ Approximately half of the known adult BMI SNPs showed nominal effects on BMI at age 8, suggesting that these SNPs begin having an effect in childhood. The SNP effect sizes on BMI at age 8 are larger than the effects on adult BMI for some SNPs; for example, the *RBJ*/*ADCY3* locus had an effect size of 0.14 kg/m^2^ in adults^8^ and 0.18 kg/m^2^ at age 8. Those SNPs that did not have an effect on BMI in childhood may indicate early-onset vs adult-onset SNPs or may be due to lower power in our study.

There are several limitations to this study. These analyses were much more computationally intensive than the cross-sectional models commonly used in GWAS.^27^ For example, both the ALSPAC and Raine GWAS took approximately 2 months (approximately 1440 h) to analyse on high performance clusters, i.e. BlueCrystal Phase 2 cluster: [https://www.acrc.bris.ac.uk/acrc/phase2.htm] and [http://www.ivec.org/], respectively. This computational burden has limited the current analyses to genetic data on the 22 autosomes using HapMap2 imputation, rather than the more recent 1000 genomes, and to two cohorts with this capacity. Replication is also challenging because of the need for detailed repeated measures data from across childhood and adolescence. Furthermore, there has been some criticism over recent years of the use of BMI as a measure of adiposity throughout childhood because at some ages BMI remains correlated with height and this correlation changes with age.^48^ Stergiakouli *et al.*^17^ showed that a different power of height was required at different ages throughout childhood, ranging from 1.5 at 12 months to 3.1 at 8–12 years. However, their results suggest that genetic influences on weight/height are similar to those for BMI adjusted for height. We therefore conducted a sensitivity analysis of our novel SNP by including a fixed effect for change in height and showed that the association between rs944990 and BMI remained.

Identifying genetic variants that have age-specific effects has the potential to shed light on the life-course aetiology of health and disease as well as potentially providing clues to gene function. Our results are consistent with the hypothesis that the genetic determinants of adult susceptibility to obesity act on both BMI intercept at 8 years and trajectory from childhood, change with age and develop over the life course. As with adult loci, the genetic variants associated with BMI in children only explain a small proportion of the estimated heritability of childhood BMI. Hence, considerable opportunity exists for new insights into the biology of childhood obesity.

## Supplementary Data

Supplementary data are available at *IJE* online.

## Funding

This work was supported by the following funding sources. N.M.W. was funded by an Australian Postgraduate Award from the Australian Government of Innovation, Industry, Science and Research and a Raine Study PhD Top-Up Scholarship. L.D.H. is supported by a UK Medical Research Fellowship (G1002375). L.D.H., L.P., G.D.S., K.T., N.J.T. and D.A.L. work in a unit that receives funding from the University of Bristol and the UK Medical Research Council (MC_UU_12013/5 and MC_UU_12013/9). University of Bristol block grant for RCUK funded research.

*ALSPAC*: the UK Medical Research Council and the Wellcome Trust (Grant ref: 102215/2/13/2) and the University of Bristol provide core support for ALSPAC.

*Raine*: The following Institutions provide funding for Core Management of the Raine Study: the University of Western Australia (UWA), Raine Medical Research Foundation, UWA Faculty of Medicine, Dentistry and Health Sciences, the Telethon Institute for Child Health Research, Curtin University, Edith Cowan University and Women and Infants Research Foundation. This study was supported by project grants from the National Health and Medical Research Council of Australia (Grant ID 403981 and ID 003209; http://www.nhmrc.gov.au/) and the Canadian Institutes of Health Research (Grant ID MOP-82893; http://www.cihr-irsc.gc.ca/e/193.html). The funders had no role in study design, data collection and analysis, decision to publish or preparation of the manuscript.

*NFBC1966*: this work was supported by the Academy of Finland (project grants 104781, 120315, 129418, Center of Excellence in Complex Disease Genetics and Public Health Challenges Research Program), University Hospital Oulu, Biocenter, University of Oulu, Finland (75617), the European Commission (EUROBLCS, Framework 5 award QLG1-CT-2000-01643), the National Heart, Lung and Blood Institute (5R01HL087679-02) through the SNP Typing for Association with Multiple Phenotypes from Existing Epidemiologic Data (STAMPEED) programme (1RL1MH083268-01), the National Institute of Health/The National Institute of Mental Health (5R01MH63706:02), European Network of Genomic and Genetic Epidemiology (ENGAGE) project and grant agreement (HEALTH-F4-2007-201413), the Medical Research Council, UK (G0500539, G0600705, PrevMetSyn/Public Health Challenges Research Program), and EU Framework Programme 7 small-scale focused research collaborative project EurHEALTHAgeing (277849).

## Supplementary Material

Supplementary Data
