# Supplementary material for: A genome-wide association study of body mass index across early life and childhood
Source: Int J Epidemiol. 2015 May 7;44(2):700–12. doi: 10.1093/ije/dyv077 (PMC4469798; doi:10.1093/ije/dyv077)
Supplement: Supplementary Data [file supp_44_2_700__index.html]

A genome-wide association study of body mass index across early life and childhood — A genome-wide association study of body mass index across early life and childhood — Supplementary Data 

# A genome-wide association study of body mass index across early life and childhood

## Supplementary Data

files

**Files in this Data Supplement:**

- Supplementary Data - docx file
