# Supplementary material for: A genome-wide association study of body mass index across early life and childhood
Source: Int J Epidemiol. 2015 May 7;44(2):700–12. doi: 10.1093/ije/dyv077 (PMC4469798; doi:10.1093/ije/dyv077)
Supplement: Supplementary Data [file supp_dyv077_dyv077BMI-GWAS-Supplementary-Clean.docx]

Supplementary Material

**Participants:**

ALSPAC: From birth to five years, length and weight measurements were extracted from health visitor records. For a random 10% of the cohort, height and weight measurements were available from eight research clinic visits, held between the ages of four months and five years of age. From age seven years upwards, all children were invited to clinics at intervals of approximately one year. Details of the measuring equipment used in the clinics is described elsewhere (1). In addition, parent-reported child height and weights were also available from the questionnaires across all ages. BMI was calculated from the weight and height measurements (weight measured in kilograms divided by height measured in metres squared; median 9 measures per person, interquartile range 5-12, range 1-29 measurements). A subset of individuals was genotyped using the Illumina HumanHap550 quad genome-wide SNP genotyping platform (Illumina Inc., San Diego, CA, USA) by 23andMe subcontracting the Wellcome Trust Sanger Institute, Cambridge, UK and the Laboratory Corporation of America (LabCorp Holdings., Burlington, NC, USA) (2) .

Raine: Weight and height were collected at follow-up clinics at average ages of 1, 2, 3, 6, 8, 10, 14 and 17 years by trained members of the research team (3). BMI was calculated from the weight and height measurements (median 6 measures per person, interquartile range 5-7, range 1-8 measurements). Genotyping was conducted using the genome-wide Illumina 660 Quad Array at the Centre for Applied Genomics, Toronto, Canada.

NFBC1966: Mothers were invited to participate in the cohort if they had estimated delivery dates falling between January 1^st^ and December 31^st^ 1966 and were followed from the 24^th^ week of gestation. Data on postnatal height and weight growth were obtained from health clinic records. On average, 12 height and weight measurements per person (interquartile range 9-15, range 1-28 measurements) were available between 1 and 17 years.

**Statistical Methods:**

Implausible height, weight and their corresponding BMI measurements (>4 standard deviations from the sex and age specific mean) were considered as outliers and were re-coded to missing.

We conducted GWAS analysis in both ALSPAC and Raine, and replication in the NFBC1966, using the following model for the j^th^ individual and at the t^th^ time-point:

|  | $log\left( {BMI}_{jt} \right)=\beta_{0}+\beta_{1}\frac{{Age}_{jt}}{1!}+\beta_{2}\frac{{{Age}_{jt}}^{2}}{2!}+\beta_{3}\frac{{{Age}_{jt}}^{3}}{3!}+\beta_{4}\frac{{{(Age}_{jt}-\kappa_{1})}_{+}^{3}}{3!}+\beta_{5}\frac{{{(Age}_{jt}-\kappa_{2})}_{+}^{3}}{3!}+\beta_{6}\frac{{{(Age}_{jt}-\kappa_{3})}_{+}^{3}}{3!}+\beta_{7}SNP+\beta_{8}SNP\frac{{Age}_{jt}}{1!}+\beta_{9}SNP\frac{{{Age}_{jt}}^{2}}{2!}+\beta_{10}SNP\frac{{{Age}_{jt}}^{3}}{3!}+\beta_{11}SNP\frac{{{(Age}_{jt}-\kappa_{1})}_{+}^{3}}{3!}+\beta_{12}SNP\frac{{{(Age}_{jt}-\kappa_{2})}_{+}^{3}}{3!}+\beta_{13}SNP\frac{{{(Age}_{jt}-\kappa_{3})}_{+}^{3}}{3!}+\beta_{14}sex+\beta_{15}sex\frac{{Age}_{jt}}{1!}+\beta_{16}sex\frac{{{Age}_{jt}}^{2}}{2!}+\beta_{17}sex\frac{{{Age}_{jt}}^{3}}{3!}+\beta_{18}sex\frac{{{(Age}_{jt}-\kappa_{1})}_{+}^{3}}{3!}+\beta_{19}sex\frac{{{(Age}_{jt}-\kappa_{2})}_{+}^{3}}{3!}+\beta_{20}sex\frac{{{(Age}_{jt}-\kappa_{3})}_{+}^{3}}{3!}+\sum_{l} \mathrm{Covariate}_{l}+b_{0j}+b_{1j}\frac{{Age}_{jt}}{1!}+b_{2j}\frac{{{Age}_{jt}}^{2}}{2!}+\varepsilon_{\mathrm{jt}}$ |  |
| --- | --- | --- |

Where:

- log is the natural log of BMI.
- Age was centred at 8 years.
- κ_k_ is the k^th^ knot and (Age - κ_k_)_+_ = 0 if Age ≤ κ_k_ and (Age - κ_k_) if Age > κ_k_, which is known as the truncated power basis that ensures smooth continuity between the time windows (4).
- sex is coded as 1 for male and 2 for female.
- Covariate_l_ is the l^th^ covariate which includes the first five principal components in Raine and NFBC1966 and a variable indicating the source of measurement in ALSPAC (i.e. questionnaire or clinic based).
- $b_{0j}$, $b_{1j}$ and $b_{2j}$ are the random effects.

A Taylor series, which is a representation of a function as an infinite sum of terms that are calculated from the values of the function’s derivatives at a single point, is used in the spline function to allow for easier convergence. A series of models were fit with knot points placed at 6-month intervals around the visually estimated turning points; the model with the lowest Akaike Information Criterion was selected. In ALSPAC and Raine, the chosen model had knot points placed at ages two, eight and twelve years and a cubic slope for each spline between the knot points (5, 6). In NFBC1966, the knot points were placed at two, ten and twelve years, again with a cubic slope for each spline. All models assumed a continuous autoregressive (order 1) correlation structure. These models allow for individual variation in growth trajectories through random effects that allow each person-specific intercepts and slopes. They also allow for the adjustment of confounders, appropriately account for the correlation structure between repeated measures within an individual and allow for incomplete data assuming data are missing at random.

As described in the methods section of the manuscript, we used three tests to describe the results of the analysis. The coefficients in model (1) that are tested in each of the tests are as follows:

1. Global test (Wald test): The null hypothesis was that the seven coefficients estimated for the SNP, $\beta_{7}$- $\beta_{13}$, are simultaneously equal to zero.
2. SNP by age interaction (Wald test): Given the spline function has multiple parameters (i.e., it is a non-linear function of time), it is necessary to use a test that summarizes the effect of each SNP on BMI growth simultaneously. The null hypothesis was that the six coefficients estimated for the SNP by spline interaction, $\beta_{8}$- $\beta_{13}$ , are simultaneously equal to zero.
3. SNP effect at age 8: The null hypothesis was that $\beta_{7}$ was equal to zero. It is interpreted as the effect of the SNP on BMI at age 8 as that is the age at which our model was centered.

Wald tests were used instead of a likelihood ratio test, as a likelihood ratio test would require two models to be conducted for each SNP, doubling the computational time.

We have previously shown that the type 1 error of the genetic effect over time in linear mixed effects models such as these may be inflated if the function for age in the fixed and random effects differs (7). To ensure that the type 1 error was kept to nominal levels, a robust standard error was calculated for each fixed effect parameter and corresponding P-Value (8), using the following formula:

|  | $\left( \mathbf{X}^{'}\mathbf{V}^{-1}\mathbf{X} \right)^{-1}\left( \sum_{i=1}^{S} \mathbf{X}_{i}^{'}\mathbf{V}_{i}^{-1}\hat{\boldsymbol{\varepsilon}_{i}}\hat{\boldsymbol{\varepsilon}_{i}^{'}}\mathbf{V}_{i}^{-1}\mathbf{X}_{i} \right){(\mathbf{X}^{'}\mathbf{V}^{-1}\mathbf{X})}^{-1}$ |  |
| --- | --- | --- |

Where:

**X** is the fixed effect regressor matrix from equation (1)

**V** is the variance of **Y** from equation (1)

$$\hat{\varepsilon_{i}}=y_{i}-\mathbf{X}_{i}\beta$$

S is the number of subjects and *i* is the *i*^th^ subject

Supplementary Figure 1: Population average BMI trajectories in females and males for each of the three cohorts.


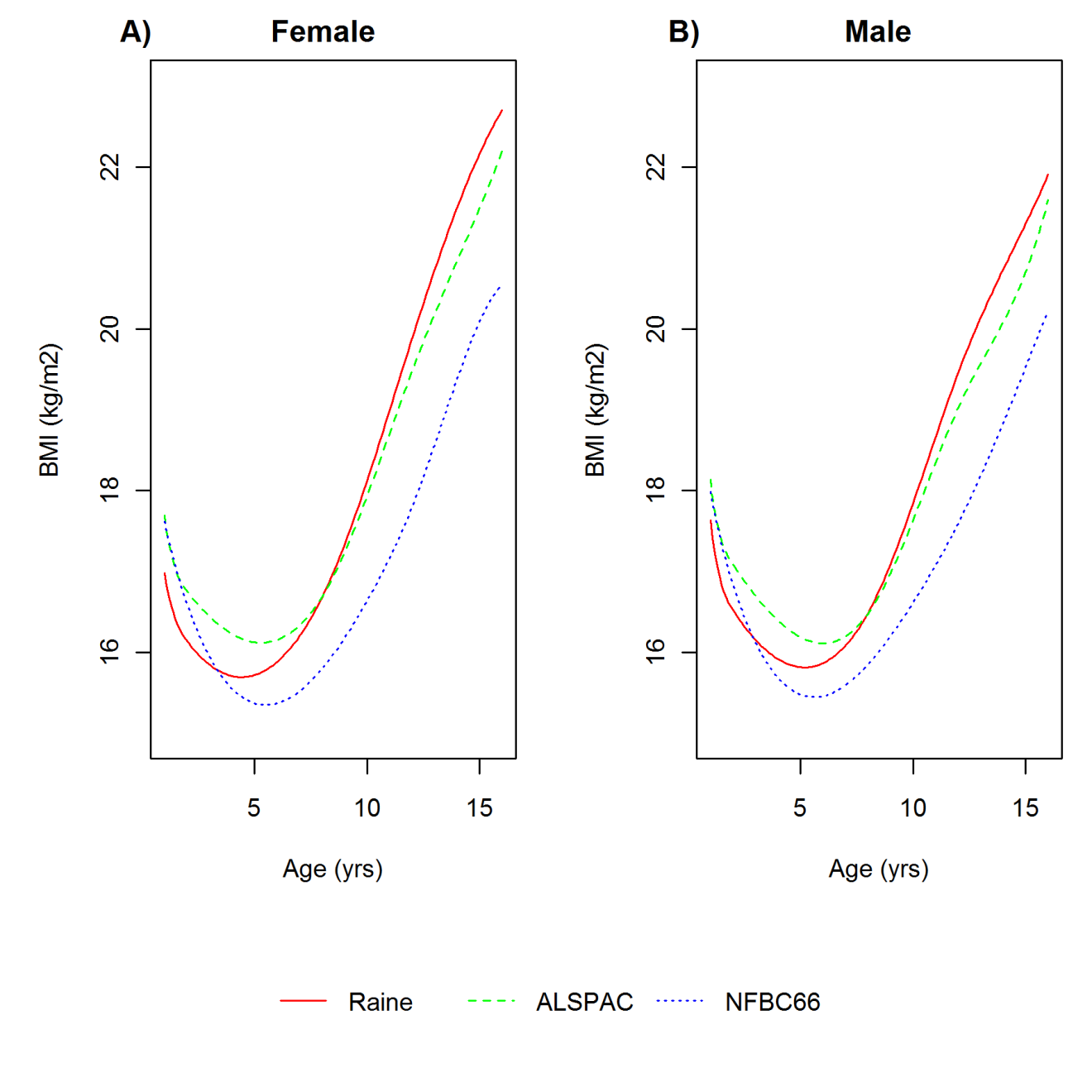


Supplementary Figure 2: QQ-plots and lambda values for the three tests from the meta-analysis of ALSPAC and Raine after GC correction has been applied.

| SNP effect at age 8  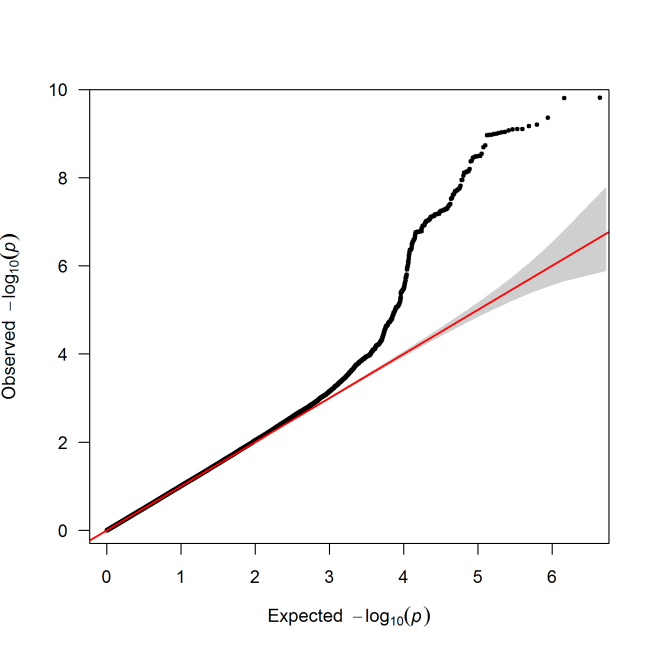  λ=1.03 | |
| --- | --- |
| Wald  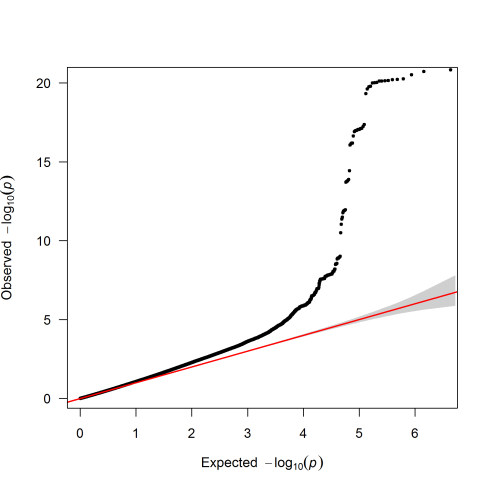  λ=1.00 | Wald (interaction)  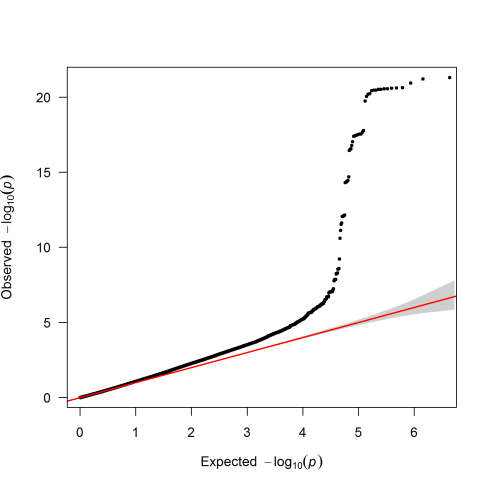  λ=1.00 |

Supplementary Figure 3: QQ-plot of the heterogeneity P-values from the fixed effects meta-analysis of the BMI intercept at age 8 in ALSPAC and Raine after GC correction has been applied.


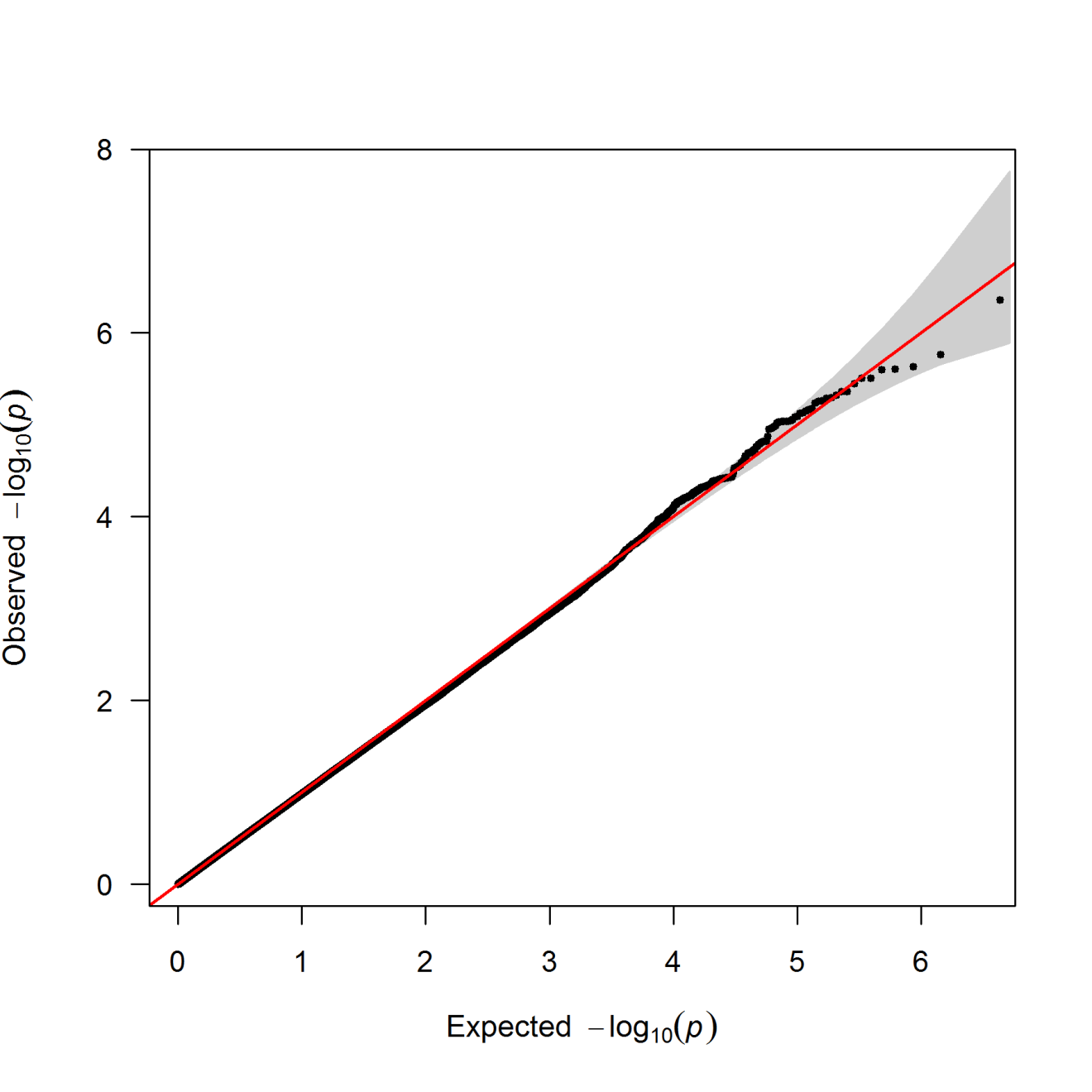


Supplementary Figure 4: Manhattan Plot for the meta-analysis of ALSPAC and Raine for the BMI intercept at age 8 with GC correction. The red line indicates genome-wide significance (5x10^-8^); the blue line indicates suggestive evidence (1x10^-5^). Gene names in black indicate loci known to be associated with adult BMI/childhood obesity; gene names in green indicate novel loci.


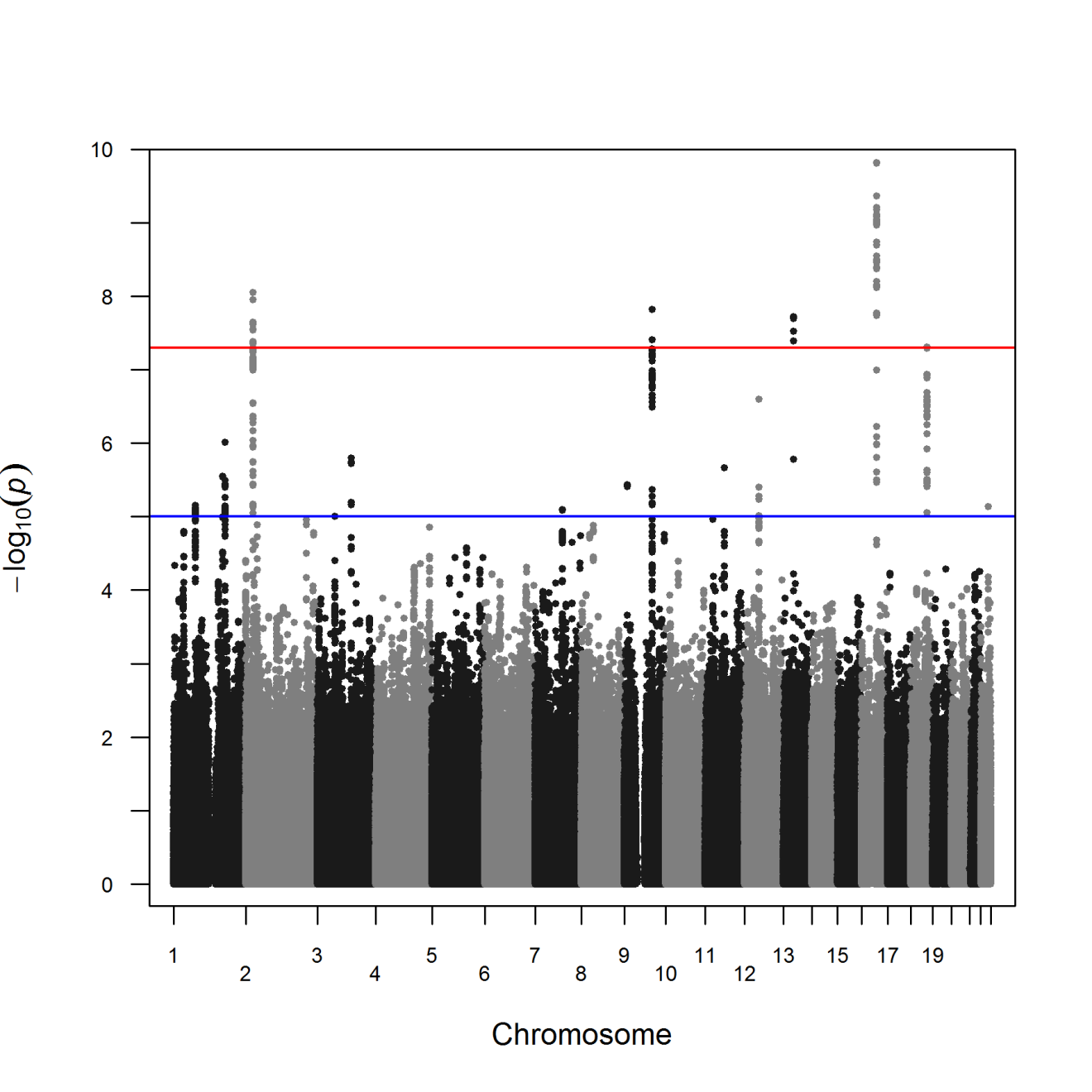


*OLFM4*

*MC4R*

*FTO*

*FAM120A/ FAM120AOS*

*ADCY3*

Supplementary Figure 5: Manhattan Plot for the meta-analysis of ALSPAC and Raine for the global Wald test with GC correction. The red line indicates genome-wide significance (5x10^-8^); the blue line indicates suggestive evidence (1x10^-5^). Gene names in black indicate loci known to be associated with adult BMI/childhood obesity.


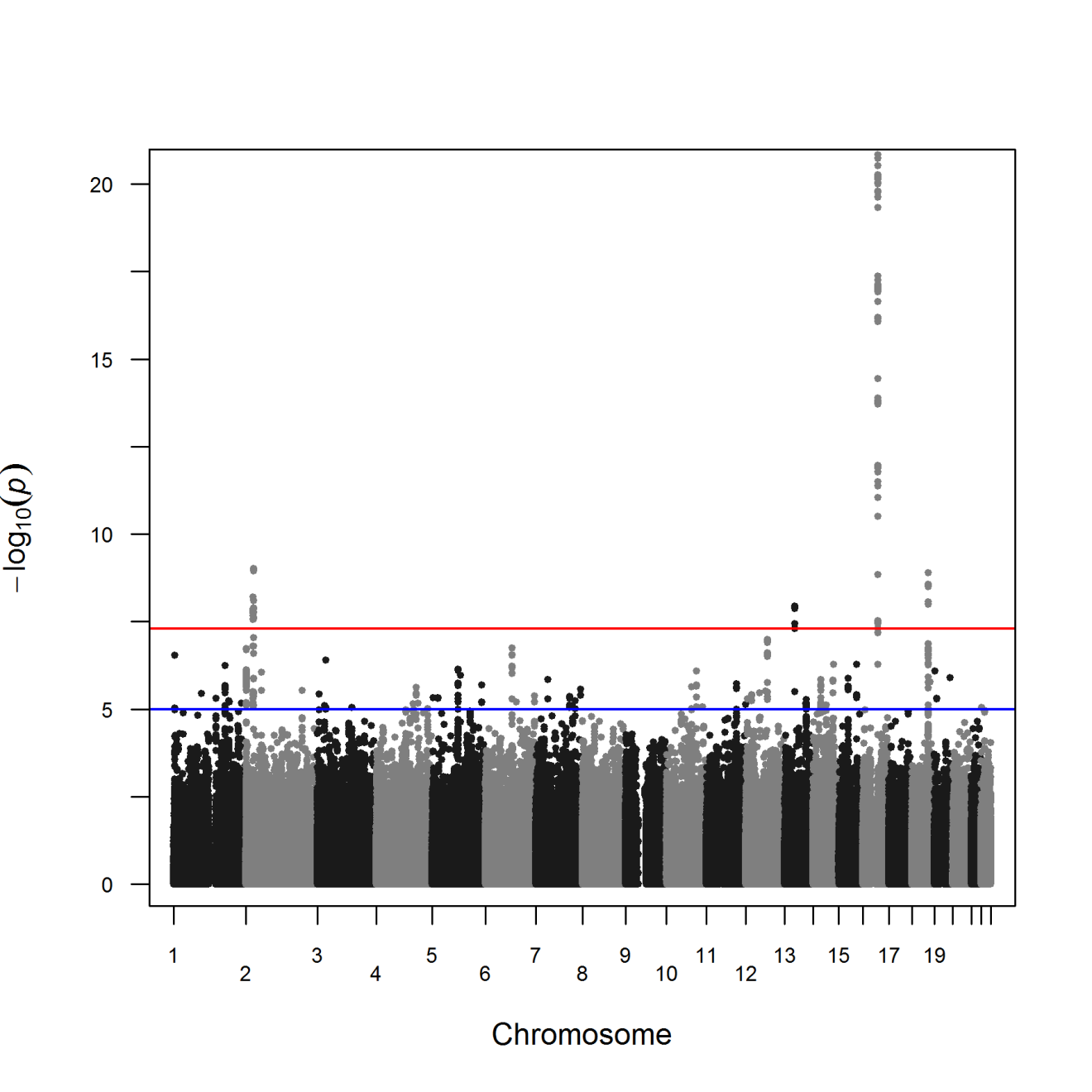


*MC4R*

*OLFM4*

*ADCY3*

*FTO*

Supplementary Figure 6: Manhattan Plot for the meta-analysis of ALSPAC and Raine for the Wald test of the SNP by age interaction with GC correction. The red line indicates genome-wide significance (5x10^-8^); the blue line indicates suggestive evidence (1x10^-5^). Gene names in black indicate loci known to be associated with adult BMI/childhood obesity.


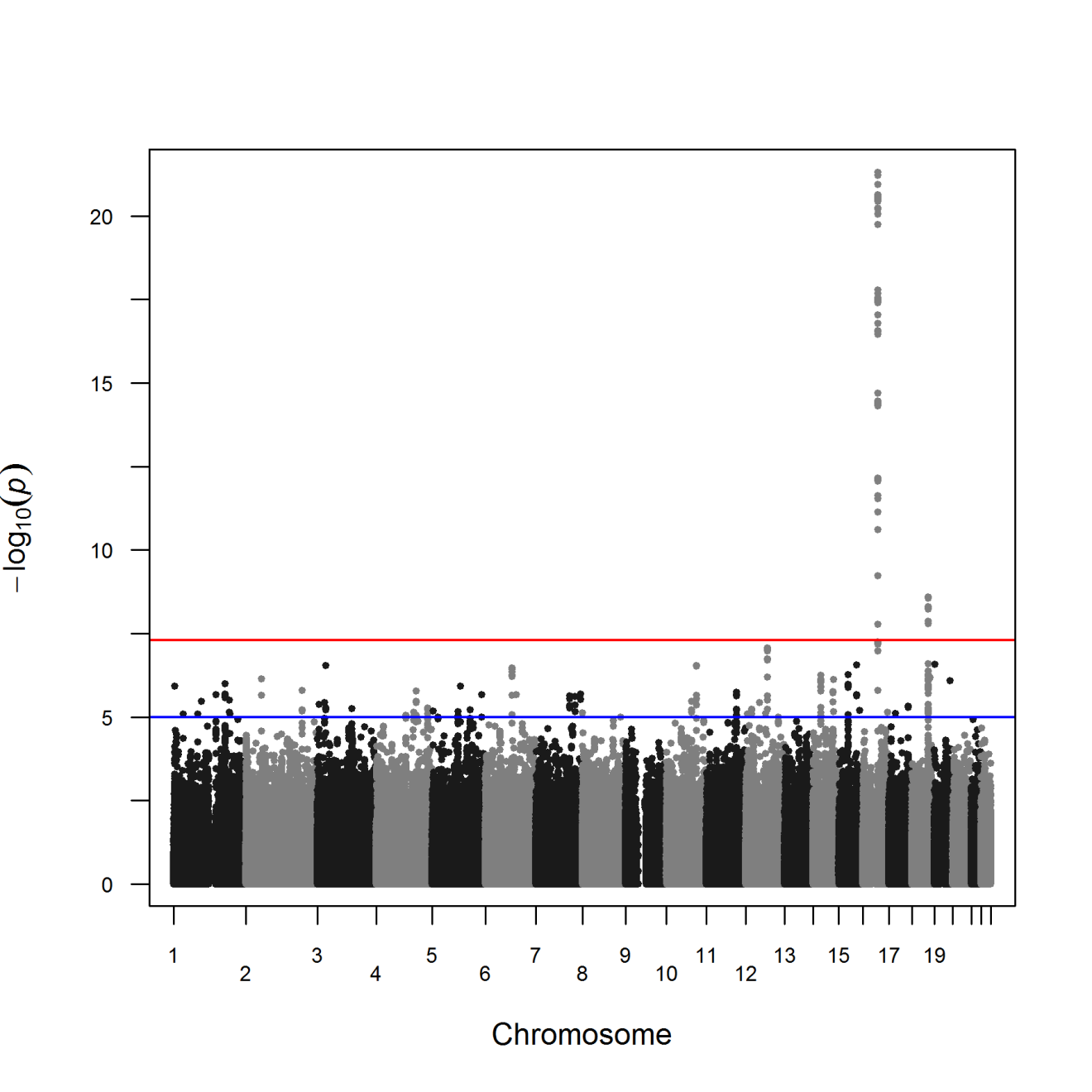


*MC4R*

*FTO*

Supplementary Figure 7: LocusZoom plots for each of the three tests at the *ADCY3* locus, rs11676272.

|  | |
| --- | --- |
|  |  |

Supplementary Figure 8: Population average trajectories for females (left) and males (right) with 0, 1 or 2 alleles at the *ADCY3*, rs11676272, locus.

| Female  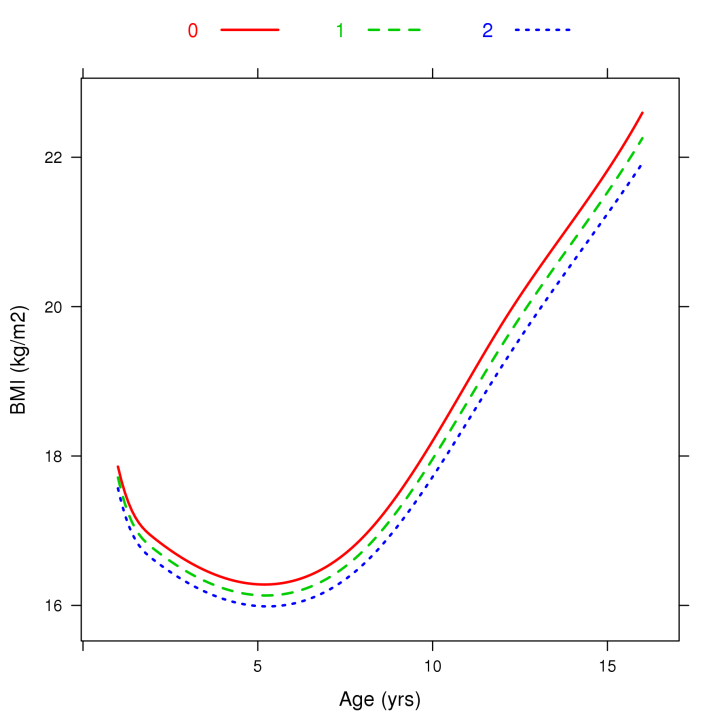 | Male  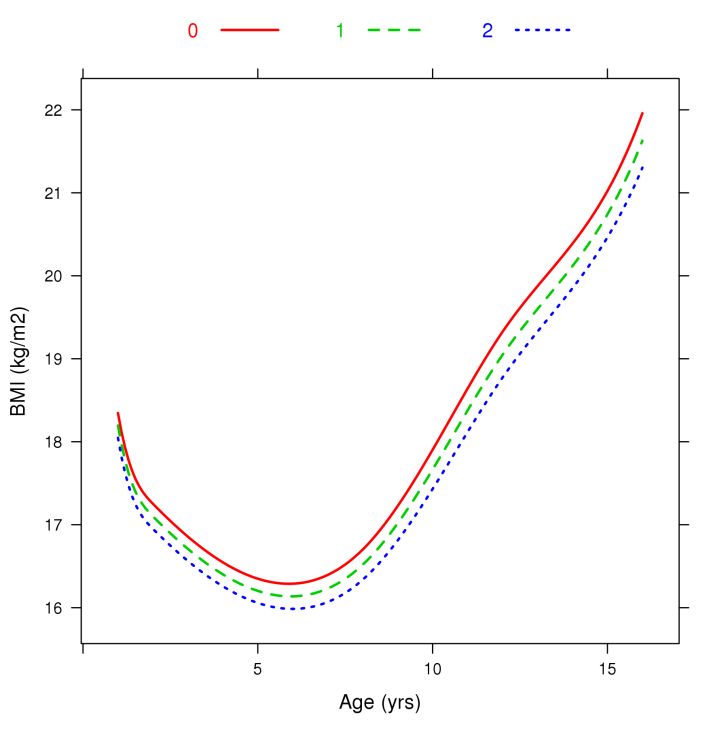 |
| --- | --- |

Supplementary Figure 9: LocusZoom plots for each of the three tests at the *FAM120AOS* locus, rs944990.

|  | |
| --- | --- |
|  |  |

Supplementary Figure 10: LocusZoom plots for each of the three tests at the *OLFM4* locus, rs12429545.

|  | |
| --- | --- |
|  |  |

Supplementary Figure 11: Population average trajectories for females (left) and males (right) with 0, 1 or 2 alleles at the *OLFM4*, rs12329545, locus

| Female  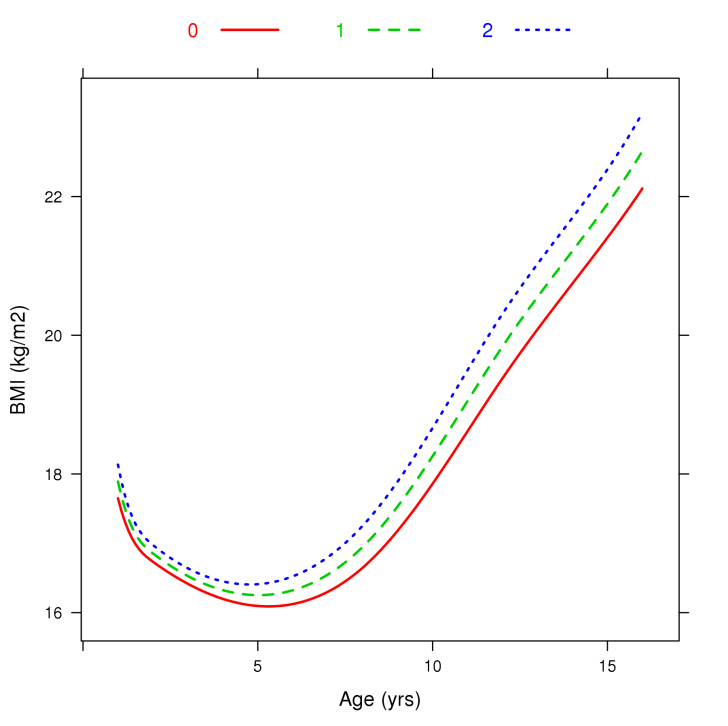 | Male  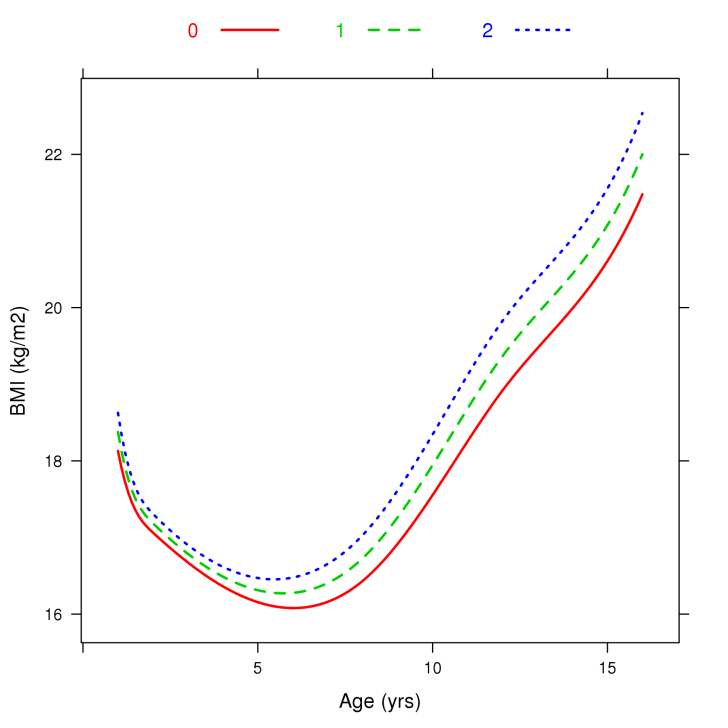 |
| --- | --- |

Supplementary Figure 12: LocusZoom plots for each of the three tests at the *FTO* locus, rs1558902.

|  | |
| --- | --- |
|  |  |

Supplementary Figure 13: Population average trajectories for females (left) and males (right) with 0, 1 or 2 alleles at the *FTO*, rs1558902, locus.

| Female  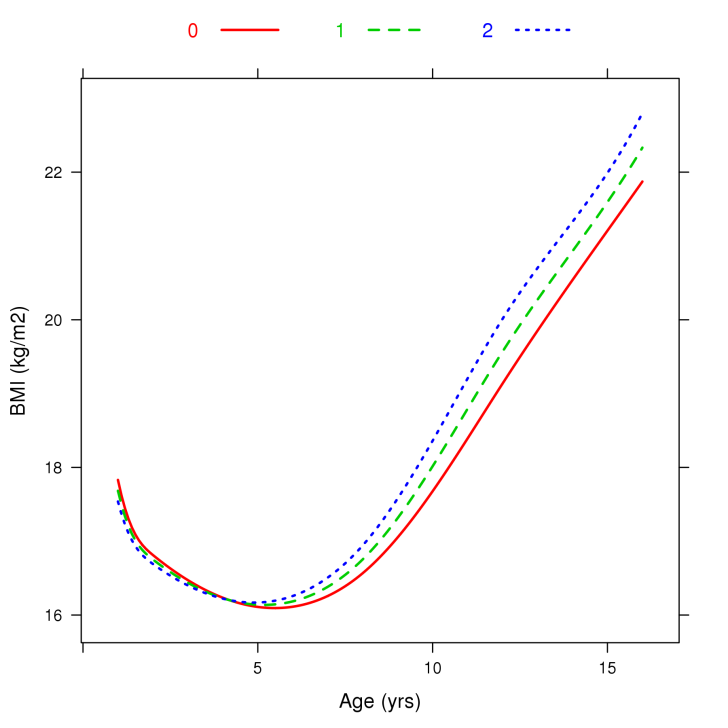 | Male  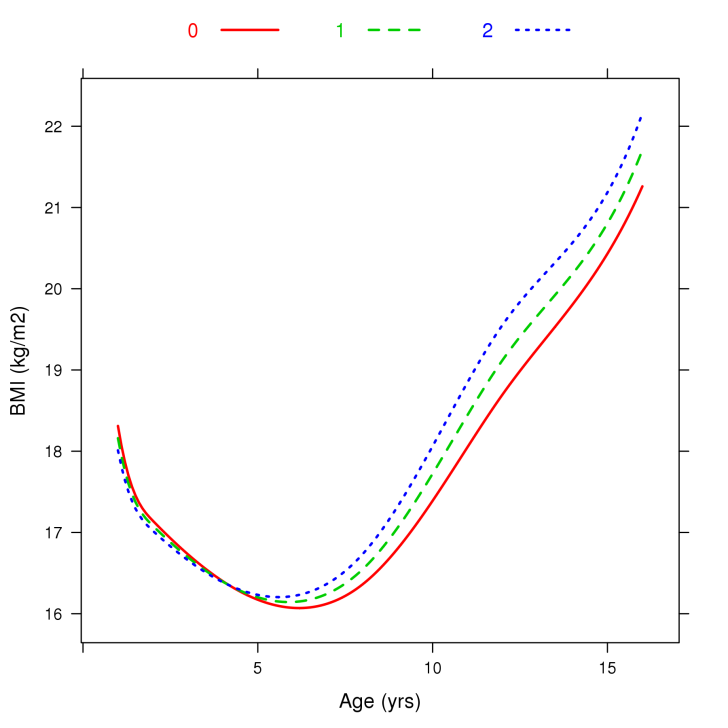 |
| --- | --- |

Supplementary Figure 14: LocusZoom plots for each of the three tests at the *MC4R* locus, rs571312.

|  | |
| --- | --- |
|  |  |

Supplementary Figure 15: Population average trajectories for females (left) and males (right) with 0, 1 or 2 alleles at the *MC4R*, rs571312, locus.

| Female  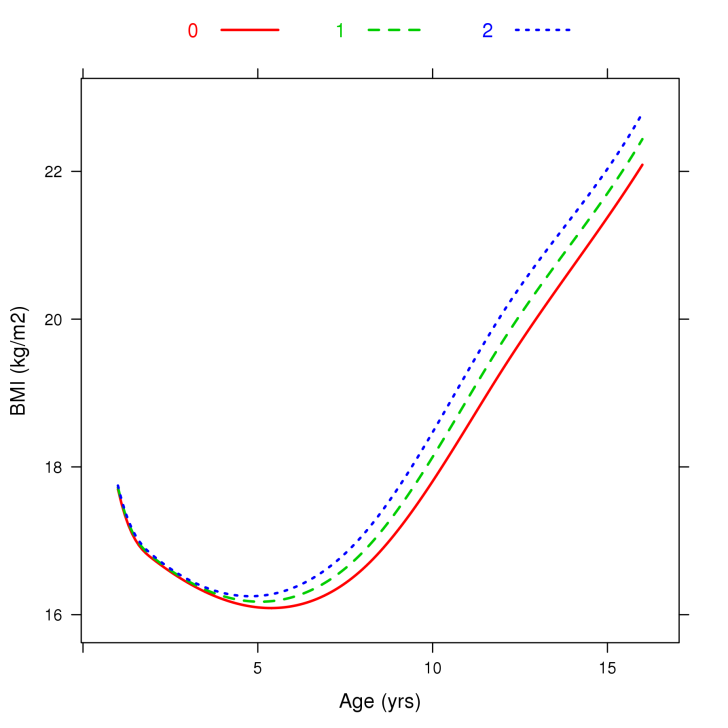 | Male  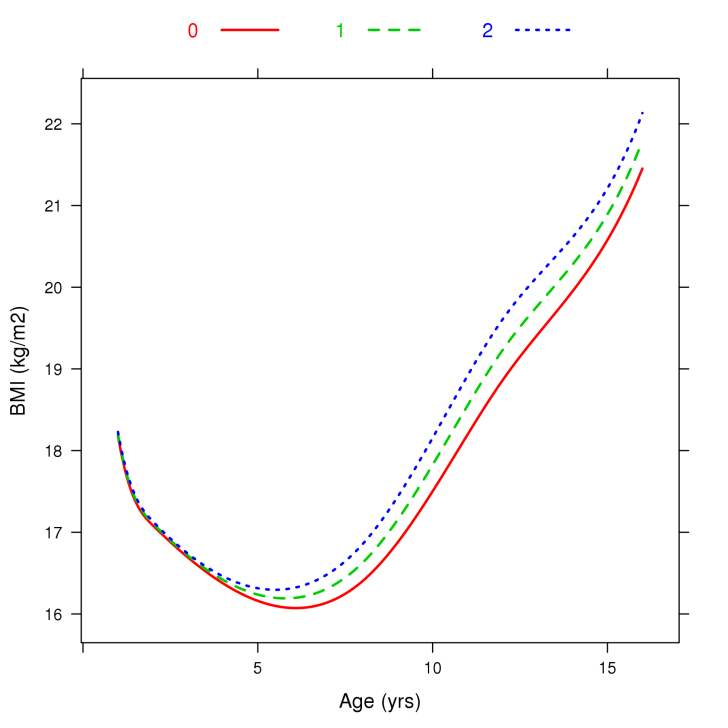 |
| --- | --- |

Supplementary Figure 16: Associations from the ALSPAC cohort between the 33 adult BMI associated SNPs and BMI from age one to 16 years. Error bars represent the regression coefficient of BMI on the natural log scale and 95% confidence intervals derived from the longitudinal additive genetic models. The SNPs are aligned to the adult BMI increasing allele.


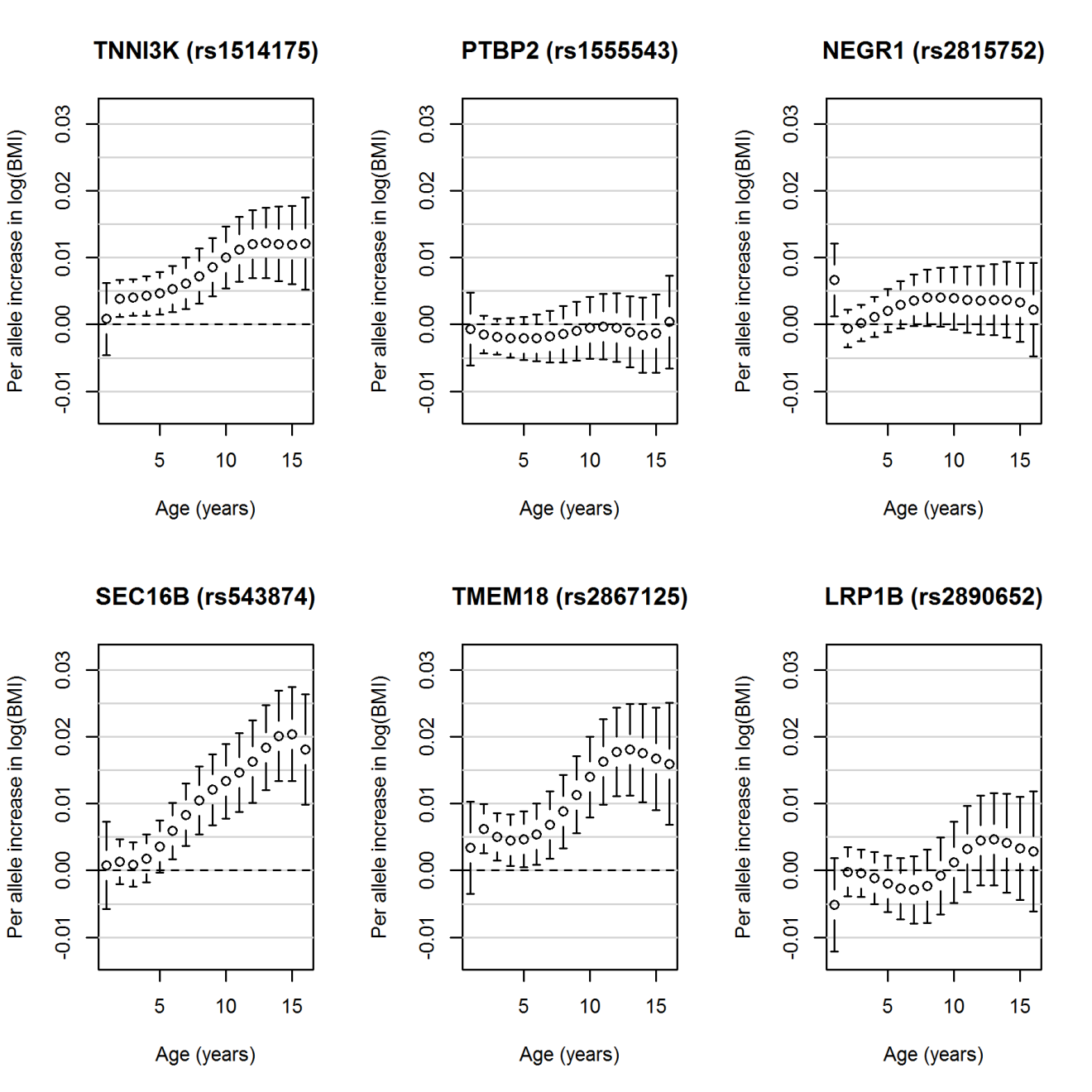


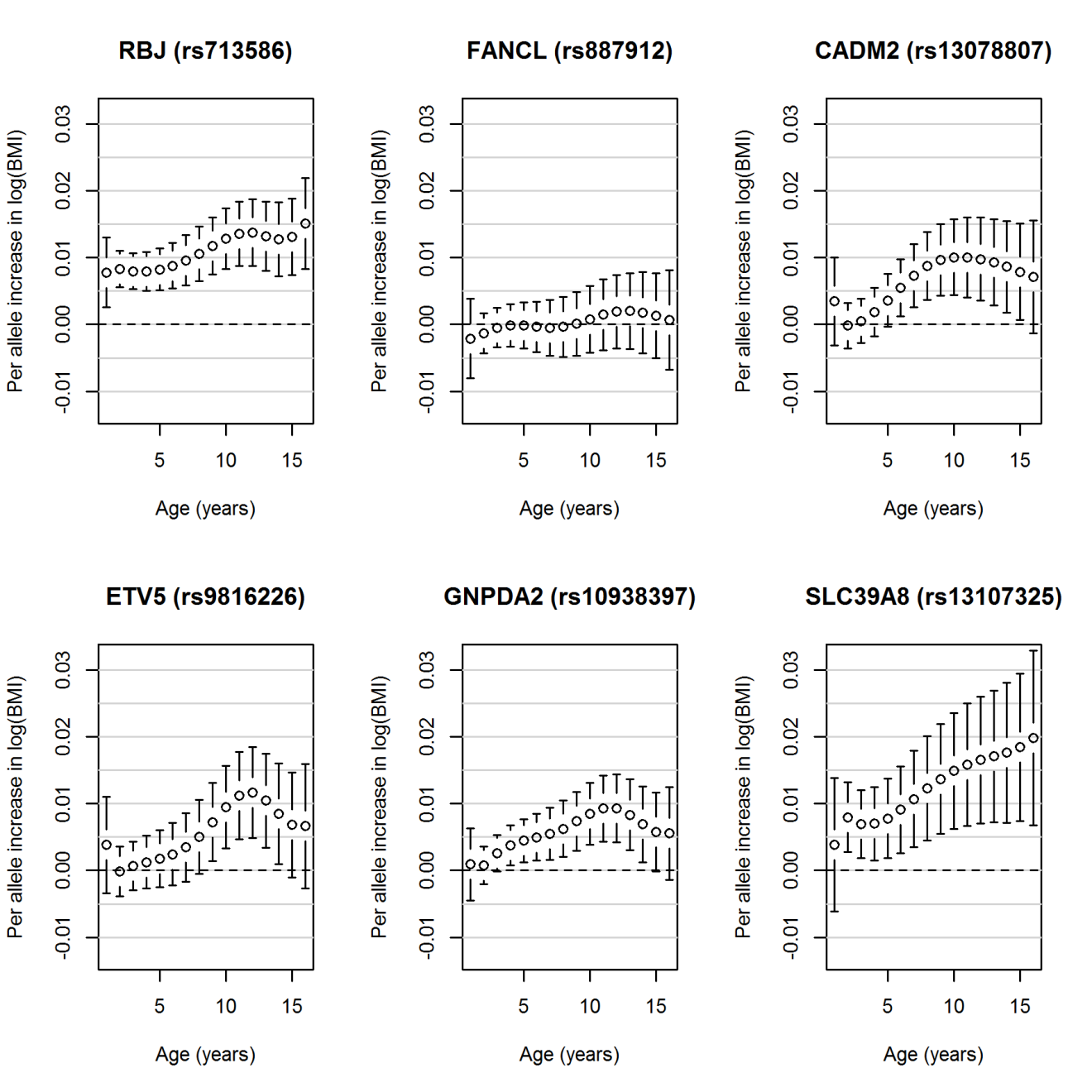


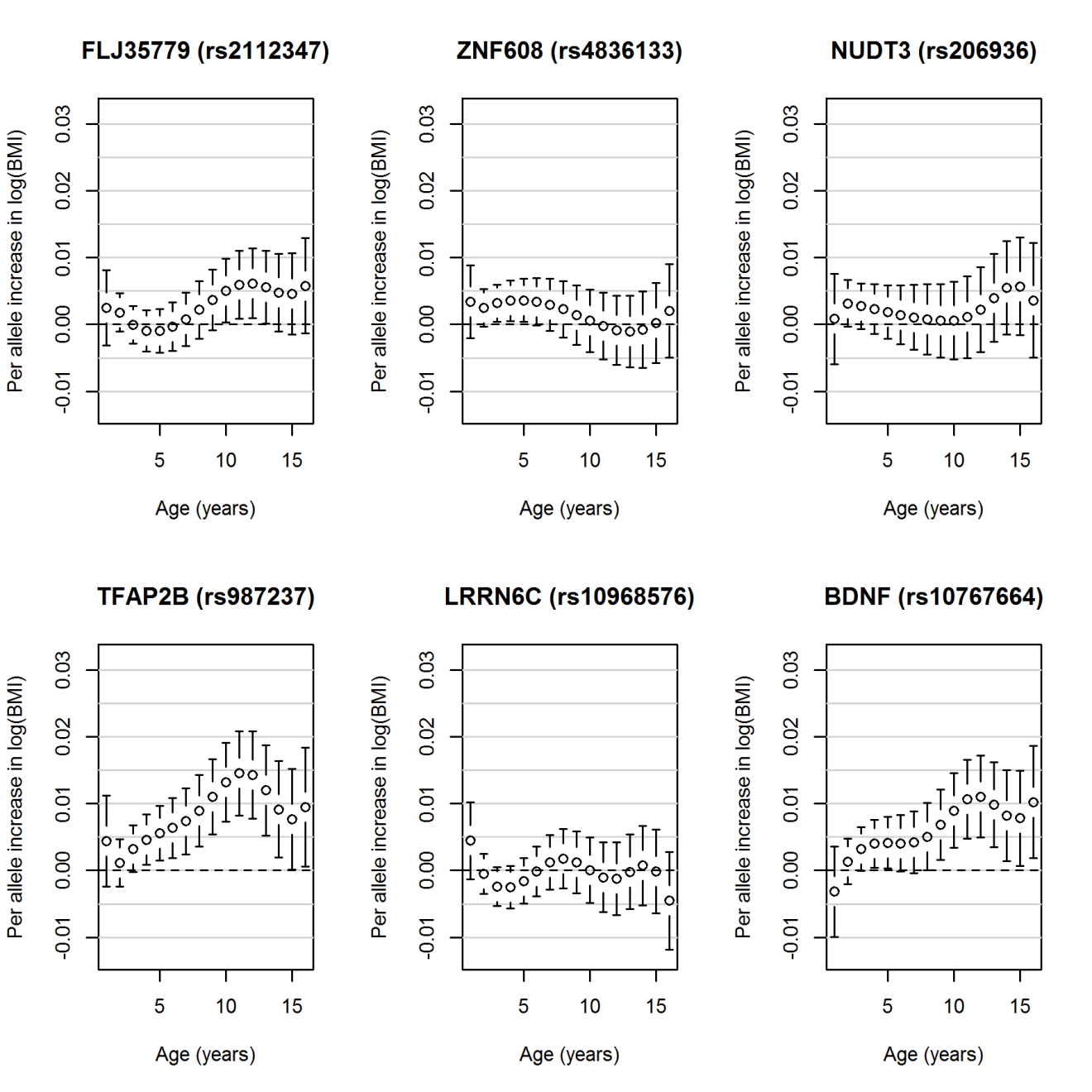


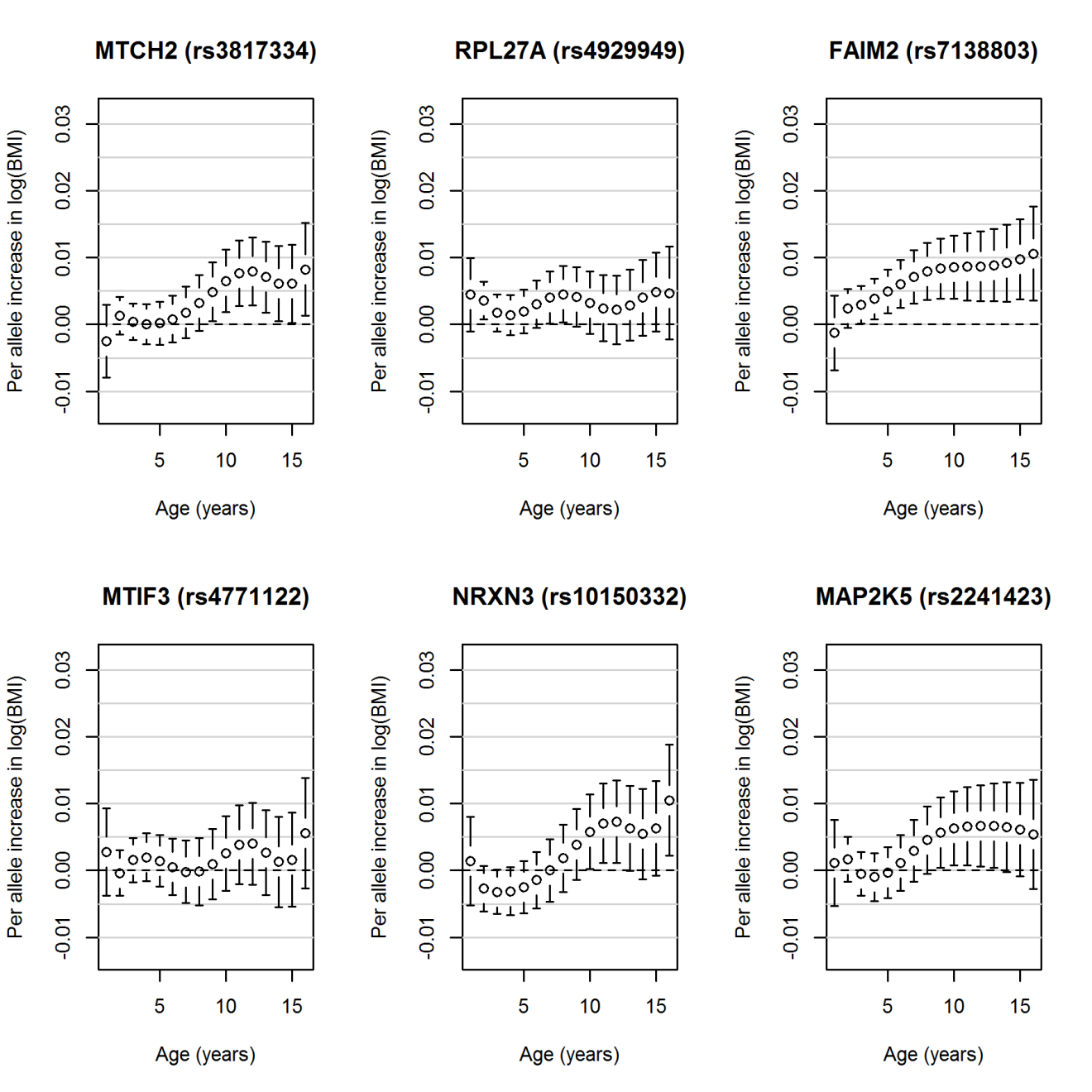


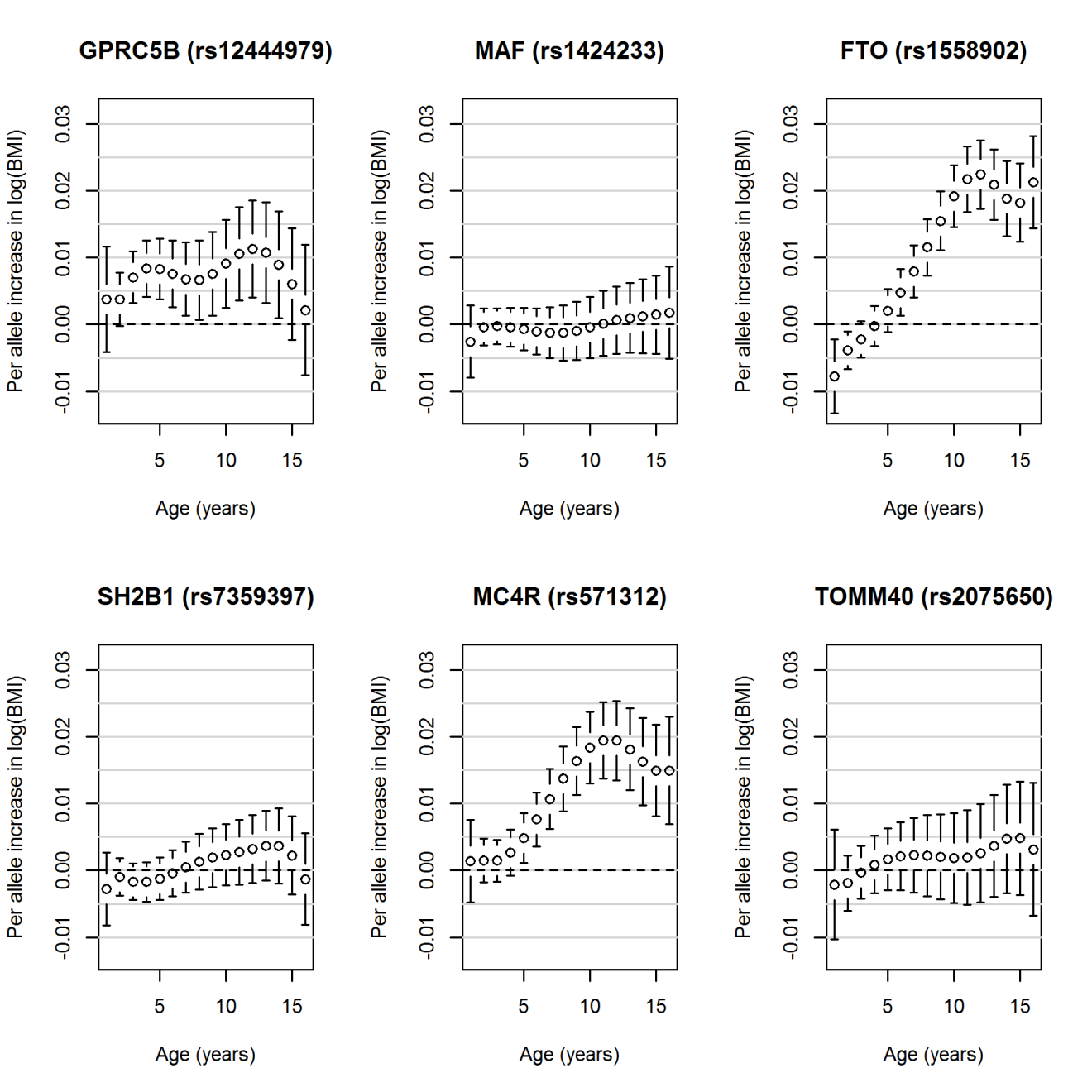


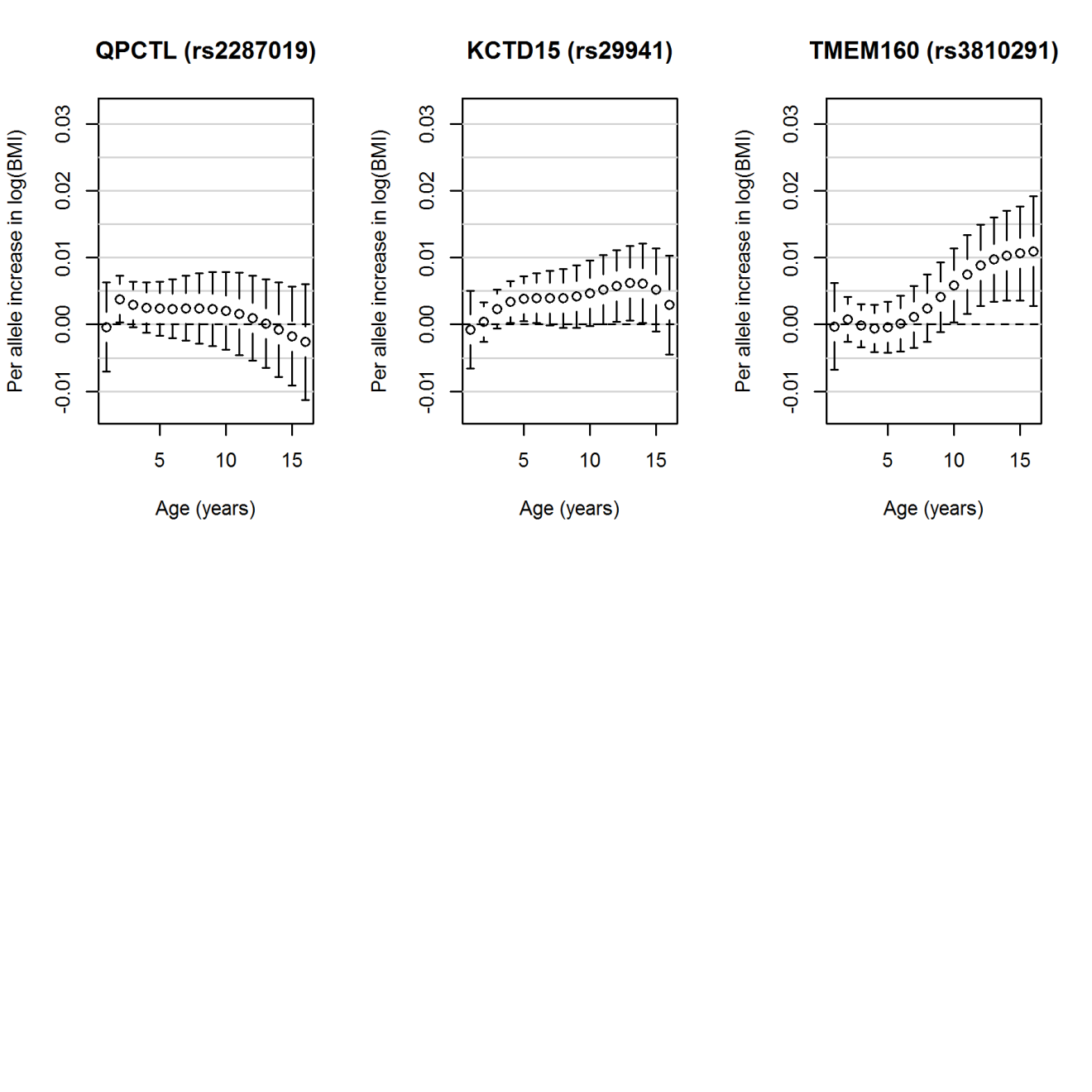


Supplementary Table 1: Results for each parameter in the spline function interacting with the most significant SNP from the genome-wide significant (P < 5x10^-8^) loci from meta-analysis. A1 is the effect allele.

|  |  |  |  |  | **ALSPAC** | | | **Raine** | | | **NFBC1966** | | |
| --- | --- | --- | --- | --- | --- | --- | --- | --- | --- | --- | --- | --- | --- |
| **Chr** | **SNP (Nearest gene)** | **A1** | **A2** |  | **Beta** | **SE** | **p** | **Beta** | **SE** | **p** | **Beta** | **SE** | **p** |
| 2 | rs11676272 (*ADCY3*) | A | G | SNP | -0.011 | 0.002 | 1.08x10^-7^ | -0.011 | 0.005 | 0.029 | -0.009 | 0.002 | 2.40x10^-4^ |
|  |  |  |  | SNP*age | -0.001 | 4.77x10^-4^ | 0.014 | -0.001 | 0.001 | 0.303 | -0.001 | 4.72x10^-4^ | 0.030 |
|  |  |  |  | SNP*age^2^ | 2.07x10^-4^ | 3.63x10^-4^ | 0.569 | 2.26x10^-4^ | 7.77x10^-4^ | 0.771 | 2.77x10^-4^ | 1.98x10^-4^ | 0.162 |
|  |  |  |  | SNP*age^3^ | 2.14x10^-5^ | 1.68x10^-4^ | 0.898 | 2.23x10^-4^ | 2.96x10^-4^ | 0.451 | 1.58x10^-4^ | 1.24x10^-4^ | 0.202 |
|  |  |  |  | SNP*age^3^ (k2) | 2.58x10^-4^ | 3.62x10^-4^ | 0.476 | -9.25x10^-5^ | 7.38x10^-4^ | 0.900 | -6.81x10^-4^ | 6.57x10^-4^ | 0.299 |
|  |  |  |  | SNP*age^3^ (k3) | -0.001 | 8.00x10^-4^ | 0.170 | -3.99x10^-4^ | 0.001 | 0.758 | 8.27x10^-4^ | 0.001 | 0.428 |
|  |  |  |  | SNP*age^3^ (k1) | -0.002 | 0.018 | 0.894 | 0.027 | 0.011 | 0.011 | -0.006 | 0.015 | 0.711 |
|  |  |  |  | Wald | -- | -- | 2.94x10^-11^ | -- | -- | 0.110 | -- | -- | 0.007 |
|  |  |  |  | Wald (interaction) | -- | -- | 0.080 | -- | -- | 0.645 | -- | -- | 0.102 |
| 9 | rs944990 (*FAM120A/ FAM120AOS*) | C | T | SNP | -0.012 | 0.002 | 4.48x10^-7^ | -0.015 | 0.006 | 0.009 | -0.004 | 0.003 | 0.109 |
|  |  |  |  | SNP*age | -0.001 | 0.001 | 0.020 | -0.002 | 0.001 | 0.108 | -1.10x10^-4^ | 5.36x10^-4^ | 0.837 |
|  |  |  |  | SNP*age^2^ | 0.001 | 3.97x10^-4^ | 0.058 | 3.78x10^-4^ | 8.30x10^-4^ | 0.649 | 2.37x10^-4^ | 2.27x10^-4^ | 0.296 |
|  |  |  |  | SNP*age^3^ | 3.71x10^-4^ | 1.87x10^-4^ | 0.048 | 1.25x10^-4^ | 3.21x10^-4^ | 0.697 | 7.87x10^-5^ | 1.44x10^-4^ | 0.585 |
|  |  |  |  | SNP*age^3^ (k2) | -5.17x10^-4^ | 3.98x10^-4^ | 0.194 | 1.93x10^-6^ | 7.95x10^-4^ | 0.998 | -9.07x10^-5^ | 7.55x10^-4^ | 0.904 |
|  |  |  |  | SNP*age^3^ (k3) | 1.05x10^-4^ | 8.88x10^-4^ | 0.906 | -7.98x10^-4^ | 0.001 | 0.564 | -2.82x10^-4^ | 0.001 | 0.813 |
|  |  |  |  | SNP*age^3^ (k1) | 0.028 | 0.020 | 0.164 | -0.028 | 0.011 | 0.012 | -0.009 | 0.017 | 0.605 |
|  |  |  |  | Wald | -- | -- | 2.93x10^-5^ | -- | -- | 0.201 | -- | -- | 0.108 |
|  |  |  |  | Wald (interaction) | -- | -- | 0.003 | -- | -- | 0.210 | -- | -- | 0.134 |
| 13 | rs12429545 (*OLFM4*) | A | G | SNP | 0.018 | 0.003 | 1.98x10^-8^ | 0.009 | 0.007 | 0.244 | 0.008 | 0.003 | 0.019 |
|  |  |  |  | SNP*age | 0.003 | 0.001 | 4.44x10^-4^ | 0.003 | 0.002 | 0.046 | 0.001 | 6.17x10^-4^ | 0.088 |
|  |  |  |  | SNP*age^2^ | -3.54x10^-4^ | 5.34x10^-4^ | 0.507 | 1.59x10^-5^ | 0.001 | 0.988 | 1.11x10^-4^ | 2.81x10^-4^ | 0.692 |
|  |  |  |  | SNP*age^3^ | -2.95x10^-4^ | 2.50x10^-4^ | 0.239 | -1.25x10^-4^ | 4.04x10^-4^ | 0.757 | 3.48x10^-5^ | 1.71x10^-4^ | 0.839 |
|  |  |  |  | SNP*age^3^ (k2) | 1.26x10^-4^ | 5.33x10^-4^ | 0.813 | 2.23x10^-5^ | 0.001 | 0.983 | -3.12x10^-4^ | 9.25x10^-4^ | 0.736 |
|  |  |  |  | SNP*age^3^ (k3) | 0.001 | 0.001 | 0.423 | -5.78x10^-5^ | 0.002 | 0.974 | 1.85x10^-4^ | 0.001 | 0.901 |
|  |  |  |  | SNP*age^3^ (k1) | 0.040 | 0.028 | 0.146 | 0.009 | 0.004 | 0.025 | 0.011 | 0.021 | 0.591 |
|  |  |  |  | Wald | -- | -- | 6.94x10^-10^ | -- | -- | 0.090 | -- | -- | 0.031 |
|  |  |  |  | Wald (interaction) | -- | -- | 1.28x10^-5^ | -- | -- | 0.069 | -- | -- | 0.051 |
| 16 | rs1558902 (*FTO*) | A | T | SNP | 0.012 | 0.002 | 7.62x10^-8^ | 0.020 | 0.005 | 1.75x10^-4^ | 0.013 | 0.002 | 2.94X10^-7^ |
|  |  |  |  | SNP*age | 0.004 | 4.78x10^-4^ | 1.15x10^-15^ | 0.002 | 0.001 | 0.021 | 0.002 | 4.70x10^-4^ | 2.18x10^-5^ |
|  |  |  |  | SNP*age^2^ | 4.94x10^-4^ | 3.64x10^-4^ | 0.175 | -0.001 | 8.01x10^-4^ | 0.194 | -5.51x10^-4^ | 2.06x10^-4^ | 0.008 |
|  |  |  |  | SNP*age^3^ | 3.36x10^-5^ | 1.71x10^-4^ | 0.844 | -4.53x10^-4^ | 3.08x10^-4^ | 0.141 | -3.01x10^-4^ | 1.27x10^-4^ | 0.018 |
|  |  |  |  | SNP*age^3^ (k2) | -8.27x10^-4^ | 3.63x10^-4^ | 0.023 | 0.001 | 0.001 | 0.349 | 7.70x10^-4^ | 6.73x10^-4^ | 0.253 |
|  |  |  |  | SNP*age^3^ (k3) | 0.003 | 0.001 | 2.97x10-4 | -3.07x10^-4^ | 0.001 | 0.814 | -4.61x10^-5^ | 0.001 | 0.965 |
|  |  |  |  | SNP*age^3^ (k1) | -0.015 | 0.019 | 0.411 | -0.040 | 0.009 | 9.57x10^-6^ | -0.006 | 0.016 | 0.688 |
|  |  |  |  | Wald | -- | -- | 4.81x10^-24^ | -- | -- | 0.010 | -- | -- | 4.48x10^-7^ |
|  |  |  |  | Wald (interaction) | -- | -- | 1.02x10^-24^ | -- | -- | 0.006 | -- | -- | 1.23x10^-6^ |
| 18 | rs571312 (*MC4R*) | A | C | SNP | 0.014 | 0.002 | 3.54x10^-8^ | 0.006 | 0.006 | 0.361 | 0.003 | 0.003 | 0.280 |
|  |  |  |  | SNP*age | 0.003 | 0.001 | 2.32x10^-7^ | 0.003 | 0.001 | 0.020 | 0.001 | 6.35x10^-4^ | 0.096 |
|  |  |  |  | SNP*age^2^ | -3.74x10^-4^ | 4.31x10^-4^ | 0.385 | 0.001 | 0.001 | 0.173 | 3.01x10^-4^ | 2.54x10^-4^ | 0.234 |
|  |  |  |  | SNP*age^3^ | -3.29x10^-4^ | 1.99x10^-4^ | 0.098 | 4.68x10^-4^ | 3.51x10^-4^ | 0.182 | 4.1010^-5^ | 1.66x10^-4^ | 0.805 |
|  |  |  |  | SNP*age^3^ (k2) | 6.32x10^-5^ | 4.26x10^-4^ | 0.882 | -0.001 | 0.001 | 0.099 | -9.81x10^-4^ | 8.45x10^-4^ | 0.240 |
|  |  |  |  | SNP*age^3^ (k3) | 0.001 | 0.001 | 0.208 | 0.003 | 0.002 | 0.088 | 0.002 | 0.001 | 0.189 |
|  |  |  |  | SNP*age^3^ (k1) | -0.010 | 0.021 | 0.619 | 0.067 | 0.006 | 6.60x10^-25^ | 0.005 | 0.021 | 0.811 |
|  |  |  |  | Wald | -- | -- | 5.10x10^-11^ | -- | -- | 0.085 | -- | -- | 0.080 |
|  |  |  |  | Wald (interaction) | -- | -- | 1.75x10^-10^ | -- | -- | 0.059 | -- | -- | 0.064 |

Supplementary Table 2: Results from association analysis between the *FAM120AOS* SNP, rs944990, and BMI trajectory, BMI adjusted for height, height trajectory and weight trajectory.

|  | **BMI** | | | **BMI adjusted for height** | | | **Height** | | |  | **Weight** | | |
| --- | --- | --- | --- | --- | --- | --- | --- | --- | --- | --- | --- | --- | --- |
|  | **Beta** | **SE** | **p** | **Beta** | **SE** | **p** | **Beta** | **SE** | **p** |  | **Beta** | **SE** | **p** |
| SNP | -0.012 | 0.002 | 4.48x10^-7^ | -0.013 | 0.002 | 1.42x10^-7^ | -0.302 | 0.107 | 0.005 | SNP | -0.016 | 0.003 | 5.71x10^-7^ |
| SNP*age | -0.001 | 0.001 | 0.020 | -0.001 | 3.89x10^-4^ | 0.022 | -0.001 | 0.017 | 0.937 | SNP*age | -6.22x10^-4^ | 5.05x10^-4^ | 0.218 |
| SNP*age^2^ | 0.001 | 3.97x10^-4^ | 0.058 | 0.001 | 2.16x10^-4^ | 0.001 | 0.010 | 0.014 | 0.473 | SNP*age^2^ | 6.25x10^-4^ | 2.24x10^-4^ | 0.005 |
| SNP*age^3^ | 3.71x10^-4^ | 1.87x10^-4^ | 0.048 | 2.81x10^-4^ | 1.23x10^-4^ | 0.023 | -0.001 | 0.006 | 0.815 | SNP*age^1^ (k1) | -2.07x10^-4^ | 1.69x10^-4^ | 0.219 |
| SNP*age^3^ (k2) | -5.17x10^-4^ | 3.98x10^-4^ | 0.194 | -4.42x10^-4^ | 1.73x10^-4^ | 0.011 | 0.005 | 0.015 | 0.748 |  |  |  |  |
| SNP*age^3^ (k3) | 1.05x10^-4^ | 8.88x10^-4^ | 0.906 | 1.49x10^-4^ | 1.49x10^-4^ | 0.320 | -0.037 | 0.042 | 0.371 |  |  |  |  |
| SNP*age^3^ (k1) | 0.028 | 0.020 | 0.164 | 0.028 | 5.27x10^-6^ | 0.000 | 0.267 | 0.641 | 0.677 |  |  |  |  |
| Wald | -- | -- | 2.93x10^-5^ | -- | -- | 5.44x10^-5^ | -- | -- | 0.003 |  | -- | -- | 1.52x10^-5^ |
| Wald (interaction) | -- | -- | 0.003 | -- | -- | 0.007 | -- | -- | 0.002 |  | -- | -- | 1.79x10^-4^ |

Supplementary Table 3: Summary of the association analysis between rs944990 and energy intake in the ALSPAC cohort. The analysis was adjusted for a measure of plausibility of reported dietary intake**

| **Mean age (months)** | **Method of dietary assessment** | **N** | **Mean difference in energy intake (kCal) per C allele of rs944990 (95% CI)** | **P value** |
| --- | --- | --- | --- | --- |
| 39 | FFQ* | 6302 | -14.28 (-21.09 to -7.48) | <0.001 |
| 54 | FFQ | 6187 | -5.54 (-13.18 to 2.09) | 0.16 |
| 81 | FFQ | 5647 | -5.42 (-16.36 to 5.52) | 0.33 |
| 90 | 3 day food diary | 5477 | -7.54 (-17.39 to 2.31) | 0.13 |
| 105 | FFQ | 5426 | -0.63 (-12.69 to 11.43) | 0.92 |
| 128 | 3 day food diary | 5625 | -12.80 (-24.35 to -1.25) | 0.03 |
| 166 | 3 day food diary | 4729 | 9.07 (-7.75 to 25.89) | 0.29 |

*Food frequency questionnaire

**For details see: Anderson EL, Tilling K, Fraser A, Macdonald-Wallis C, Emmett P, Cribb V, Northstone K, Lawlor DA, Howe LD. Estimating trajectories of energy intake through childhood and adolescence using linear-spline multilevel models. Epidemiology. 2013 Jul;24(4):507-15. doi: 10.1097/EDE.0b013e318295af33

Supplementary Table 4: Association results for rs944990 in *FAM120AOS* from publically available genome-wide association studies for relevant endpoints.

| Phenotype | Consortium | P-Value rs944990 | P-Value rs10821128 |
| --- | --- | --- | --- |
| BMI in adults (mean) | GIANT (9) (http://www.broadinstitute.org/collaboration/giant/index.php/GIANT_consortium) | 0.887 | 0.931 |
| BMI in adults (variance) | GIANT (10) (http://www.broadinstitute.org/collaboration/giant/index.php/GIANT_consortium) | 0.063 | 0.092 |
| Height in adults | GIANT (11) (http://www.broadinstitute.org/collaboration/giant/index.php/GIANT_consortium) | 0.889 | 0.731 |
|  | GIANT (12) (http://www.broadinstitute.org/collaboration/giant/index.php/GIANT_consortium) | 0.43 | 0.53 |
| Waist hip ratio in adults (adjusted for BMI) | GIANT (13) (http://www.broadinstitute.org/collaboration/giant/index.php/GIANT_consortium) | 0.97 | 0.93 |
| Obesity in adults (class 1) | GIANT (14) (http://www.broadinstitute.org/collaboration/giant/index.php/GIANT_consortium) | 0.65 | 0.23 |
| Obesity in adults (class 2) |  | 0.15 | 0.35 |
| Obesity in adults (class 3) |  | 0.78 | 0.98 |
| Fasting glucose in adults | MAGIC (15) (http://www.magicinvestigators.org/) | 0.936 | 0.601 |
| Fasting insulin in adults |  | 0.957 | 0.814 |
| Fasting HOMA-IR in adults |  | 0.883 | 0.838 |
| Type 2 Diabetes | Diagram (16) (http://diagram-consortium.org/index.html) | 0.26 | 0.67 |
| Age of Menarche | Reprogen (17) (http://www.reprogen.org/) | 4.0x10^-4^ | 5.9x10^-4^ |
| Obesity in children | EGG (18) (http://egg-consortium.org/) | 0.390 | 0.353 |
| Birth weight | EGG (19) (http://egg-consortium.org/) | 0.23 | 0.28 |
| Birth length | EGG (20) (http://egg-consortium.org/) | 0.526 | 0.682 |
| Head circumference | EGG (21) (http://egg-consortium.org/) | 0.098 | 0.009 |
| Pubertal growth – “Take-off phase of growth spurt” | EGG (22) (http://egg-consortium.org/) | 0.758 | 0.291 |
| Pubertal growth – total amount of growth across pubertal growth period | EGG (22) (http://egg-consortium.org/) | 0.140 | 0.044 |
| Pubertal growth – growth in late adolescence | EGG (22) (http://egg-consortium.org/) | 0.972 | 0.796 |

Supplementary Table 5: Results from the three tests investigated in the meta-analysis for the 33 SNPs associated with adult BMI.

|  |  |  |  | **Alleles** | |  |  | **Per allele change in log(BMI)** |  | **Per allele change in log(BMI)** |  |  |  |
| --- | --- | --- | --- | --- | --- | --- | --- | --- | --- | --- | --- | --- | --- |
| **SNP** | **Nearest**  **Gene** | **Chr.** | **Position^a^ (bp)** | **Effect^b^** | **Other** | **Effect Allele Frequency^c^** |  | **ALSPAC**  **β (S.E.)** | **ALSPAC P-value** | **Raine**  **β (S.E.)** | **Raine P-value** | **Meta-Analysis P-value** | **Het I^2^ (P-value)** |
| rs2815752 | *NEGR1* | 1 | 72585028 | A | G | 0.601 | SNP | 0.004 (0.002) | 0.062 | 0.008 (0.005) | 0.117 | 0.020 | 0 (0.462) |
|  |  |  |  |  |  |  | Wald |  | 0.080 |  | 0.065 | 0.057 |  |
|  |  |  |  |  |  |  | Wald (interaction) |  | 0.064 |  | 0.056 | 0.044 |  |
| rs1514175 | *TNNI2K* | 1 | 74764232 | A | G | 0.429 | SNP | 0.007 (0.002) | 6.39x10^-4^ | 0.014 (0.005) | 0.005 | 2.30x10^-5^ | 40.3 (0.196) |
|  |  |  |  |  |  |  | Wald |  | 4.07x10^-4^ |  | 0.078 | 5.75x10^-4^ |  |
|  |  |  |  |  |  |  | Wald (interaction) |  | 0.015 |  | 0.103 | 0.015 |  |
| rs1555543 | *PTBP2* | 1 | 96717385 | C | A | 0.593 | SNP | -0.001 (0.002) | 0.496 | 0.012 (0.005) | 0.020 | 0.803 | 82.7 (0.016) |
|  |  |  |  |  |  |  | Wald |  | 0.819 |  | 0.008 | 0.547 |  |
|  |  |  |  |  |  |  | Wald (interaction) |  | 0.898 |  | 0.005 | 0.647 |  |
| rs543874 | *SEC16B* | 1 | 176156103 | G | A | 0.207 | SNP | 0.010 (0.003) | 4.81x10^-5^ | 0.011 (0.006) | 0.068 | 8.41x10^-6^ | 0 (0.897) |
|  |  |  |  |  |  |  | Wald |  | 2.98x10^-7^ |  | 0.122 | 2.20x10^-6^ |  |
|  |  |  |  |  |  |  | Wald (interaction) |  | 4.30x10^-7^ |  | 0.076 | 2.07x10^-6^ |  |
| rs2867125 | *TMEM18* | 2 | 612827 | C | T | 0.832 | SNP | 0.009 (0.003) | 0.002 | 0.012 (0.007) | 0.068 | 3.00x10^-4^ | 0 (0.639) |
|  |  |  |  |  |  |  | Wald |  | 1.27x10^-7^ |  | 0.154 | 1.45x10^-6^ |  |
|  |  |  |  |  |  |  | Wald (interaction) |  | 1.35x10^-5^ |  | 0.154 | 6.75x10^-5^ |  |
| rs713586 | *RBJ* | 2 | 25011512 | C | T | 0.489 | SNP | 0.011 (0.002) | 4.09x10^-7^ | 0.012 (0.005) | 0.019 | 2.39x10^-8^ | 0 (0.809) |
|  |  |  |  |  |  |  | Wald |  | 7.82x10^-10^ |  | 0.095 | 1.3310^-8^ |  |
|  |  |  |  |  |  |  | Wald (interaction) |  | 0.130 |  | 0.700 | 0.293 |  |
| rs887912 | *FANCL* | 2 | 59156381 | T | C | 0.289 | SNP | -0.0004 (0.002) | 0.875 | -0.0004 (0.006) | 0.949 | 0.866 | 0 (0.999) |
|  |  |  |  |  |  |  | Wald |  | 0.837 |  | 0.223 | 0.792 |  |
|  |  |  |  |  |  |  | Wald (interaction) |  | 0.816 |  | 0.157 | 0.740 |  |
| rs2890652 | *LRP1B* | 2 | 142676401 | C | T | 0.167 | SNP | -0.002 (0.003) | 0.405 | -0.003 (0.007) | 0.624 | 0.340 | 0 (0.886) |
|  |  |  |  |  |  |  | Wald |  | 0.024 |  | 0.141 | 0.028 |  |
|  |  |  |  |  |  |  | Wald (interaction) |  | 0.016 |  | 0.287 | 0.032 |  |
| rs13078807 | *CADM2* | 3 | 85966840 | G | A | 0.204 | SNP | 0.009 (0.003) | 8.05x10^-4^ | 0.001 (0.006) | 0.827 | 0.002 | 16.2 (0.275) |
|  |  |  |  |  |  |  | Wald |  | 0.011 |  | 0.015 | 0.004 |  |
|  |  |  |  |  |  |  | Wald (interaction) |  | 0.010 |  | 0.009 | 0.004 |  |
| rs9816226 | *ETV5* | 3 | 187317193 | T | A | 0.825 | SNP | 0.005 (0.003) | 0.076 | 0.004 (0.007) | 0.534 | 0.0608 | 0 (0.916) |
|  |  |  |  |  |  |  | Wald |  | 0.002 |  | 0.640 | 0.018 |  |
|  |  |  |  |  |  |  | Wald (interaction) |  | 0.001 |  | 0.820 | 0.022 |  |
| rs10938397 | *GNPDA2* | 4 | 44877284 | G | A | 0.434 | SNP | 0.006 (0.002) | 0.003 | 0.005 (0.005) | 0.288 | 0.002 | 0 (0.865) |
|  |  |  |  |  |  |  | Wald |  | 0.014 |  | 0.103 | 0.014 |  |
|  |  |  |  |  |  |  | Wald (interaction) |  | 0.022 |  | 0.073 | 0.018 |  |
| rs13107325 | *SLC39A8* | 4 | 103407732 | T | C | 0.076 | SNP | 0.012 (0.004) | 0.002 | 0.006 (0.010) | 0.559 | 0.002 | 0 (0.531) |
|  |  |  |  |  |  |  | Wald |  | 0.009 |  | 0.575 | 0.039 |  |
|  |  |  |  |  |  |  | Wald (interaction) |  | 0.284 |  | 0.769 | 0.509 |  |
| rs2112347 | *FLJ35779* | 5 | 75050998 | T | G | 0.638 | SNP | 0.002 (0.002) | 0.318 | 0.002 (0.005) | 0.734 | 0.292 | 0 (0.949) |
|  |  |  |  |  |  |  | Wald |  | 0.002 |  | 0.806 | 0.027 |  |
|  |  |  |  |  |  |  | Wald (interaction) |  | 0.002 |  | 0.713 | 0.017 |  |
| rs4836133 | *ZNF608* | 5 | 124360002 | A | C | 0.486 | SNP | 0.002 (0.002) | 0.295 | 0.004 (0.005) | 0.453 | 0.211 | 0 (0.764) |
|  |  |  |  |  |  |  | Wald |  | 0.064 |  | 0.680 | 0.179 |  |
|  |  |  |  |  |  |  | Wald (interaction) |  | 0.183 |  | 0.575 | 0.316 |  |
| rs206936 | *NUDT3* | 6 | 34410847 | G | A | 0.195 | SNP | 0.001 (0.003) | 0.776 | 0.003 (0.006) | 0.696 | 0.681 | 0 (0.802) |
|  |  |  |  |  |  |  | Wald |  | 0.226 |  | 0.895 | 0.525 |  |
|  |  |  |  |  |  |  | Wald (interaction) |  | 0.308 |  | 0.824 | 0.564 |  |
| rs987237 | *TFAP2B* | 6 | 50911009 | G | A | 0.178 | SNP | 0.009 (0.003) | 0.001 | 0.016 (0.007) | 0.014 | 7.75x10^-5^ | 6.3 (0.302) |
|  |  |  |  |  |  |  | Wald |  | 9.88x10^-5^ |  | 0.340 | 7.16x10^-4^ |  |
|  |  |  |  |  |  |  | Wald (interaction) |  | 2.24x10^-4^ |  | 0.715 | 0.005 |  |
| rs10968576 | *LRRN6C* | 9 | 28404339 | G | A | 0.317 | SNP | 0.002 (0.002) | 0.431 | -0.003 (0.006) | 0.653 | 0.574 | 0 (0.477) |
|  |  |  |  |  |  |  | Wald |  | 0.005 |  | 0.743 | 0.038 |  |
|  |  |  |  |  |  |  | Wald (interaction) |  | 0.002 |  | 0.645 | 0.019 |  |
| rs4929949 | *RPL27A* | 11 | 8561169 | C | T | 0.540 | SNP | 0.005 (0.002) | 0.036 | 0.005 (0.005) | 0.304 | 0.020 | 0 (0.880) |
|  |  |  |  |  |  |  | Wald |  | 0.011 |  | 0.536 | 0.042 |  |
|  |  |  |  |  |  |  | Wald (interaction) |  | 0.079 |  | 0.420 | 0.141 |  |
| rs10767664 | BDNF | 11 | 27682562 | A | T | 0.793 | SNP | 0.005 (0.003) | 0.049 | 0.013 (0.006) | 0.037 | 0.009 | 26.3 (0.244) |
|  |  |  |  |  |  |  | Wald |  | 0.008 |  | 0.043 | 0.005 |  |
|  |  |  |  |  |  |  | Wald (interaction) |  | 0.008 |  | 0.046 | 0.006 |  |
| rs3817334 | *MTCH2* | 11 | 47607569 | T | C | 0.405 | SNP | 0.003 (0.002) | 0.135 | 0.001 (0.005) | 0.875 | 0.150 | 0 (0.672) |
|  |  |  |  |  |  |  | Wald |  | 0.003 |  | 0.976 | 0.086 |  |
|  |  |  |  |  |  |  | Wald (interaction) |  | 0.002 |  | 0.973 | 0.064 |  |
| rs7138803 | *FAIM2* | 12 | 48533735 | A | G | 0.359 | SNP | 0.008 (0.002) | 2.93x10^-4^ | 0.012 (0.005) | 0.019 | 2.15x10^-5^ | 0 (0.455) |
|  |  |  |  |  |  |  | Wald |  | 0.034 |  | 0.084 | 0.029 |  |
|  |  |  |  |  |  |  | Wald (interaction) |  | 0.158 |  | 0.051 | 0.102 |  |
| rs4771122 | *MTIF3* | 13 | 26918180 | G | A | 0.233 | SNP | -0.0002 (0.003) | 0.944 | 0.028 (0.006) | 2.04x10^-5^ | 0.130 | 93.7 (6.71x10^-5^) |
|  |  |  |  |  |  |  | Wald |  | 0.109 |  | 1.06x10^-4^ | 0.011 |  |
|  |  |  |  |  |  |  | Wald (interaction) |  | 0.070 |  | 2.32x10^-4^ | 0.008 |  |
| rs10150332 | *NRXN3* | 14 | 79006717 | C | T | 0.215 | SNP | 0.002 (0.003) | 0.479 | -0.004 (0.006) | 0.537 | 0.682 | 0 (0.398) |
|  |  |  |  |  |  |  | Wald |  | 5.85x10^-4^ |  | 0.031 | 4.22x10^-4^ |  |
|  |  |  |  |  |  |  | Wald (interaction) |  | 4.12x10^-4^ |  | 0.017 | 2.30x10^-4^ |  |
| rs2241423 | *MAP2K5* | 15 | 65873892 | G | A | 0.784 | SNP | 0.005 (0.003) | 0.076 | 0.009 (0.006) | 0.145 | 0.027 | 0 (0.522) |
|  |  |  |  |  |  |  | Wald |  | 0.011 |  | 0.123 | 0.013 |  |
|  |  |  |  |  |  |  | Wald (interaction) |  | 0.008 |  | 0.092 | 0.009 |  |
| rs12444979 | *GPRC5B* | 16 | 19841101 | C | T | 0.863 | SNP | 0.007 (0.003) | 0.029 | 0.015 (0.007) | 0.036 | 0.005 | 14.8 (0.279) |
|  |  |  |  |  |  |  | Wald |  | 5.72x10^-4^ |  | 0.191 | 0.002 |  |
|  |  |  |  |  |  |  | Wald (interaction) |  | 0.006 |  | 0.138 | 0.009 |  |
| rs7359397 | *SH2B1* | 16 | 28793160 | T | C | 0.409 | SNP | 0.001 (0.002) | 0.537 | 0.007 (0.005) | 0.161 | 0.272 | 11.9 (0.287) |
|  |  |  |  |  |  |  | Wald |  | 0.098 |  | 0.884 | 0.331 |  |
|  |  |  |  |  |  |  | Wald (interaction) |  | 0.075 |  | 0.835 | 0.259 |  |
| rs1558902 | *FTO* | 16 | 52361075 | A | T | 0.402 | SNP | 0.012 (0.002) | 7.62x10^-8^ | 0.020 (0.005) | 1.75x10^-4^ | 1.54x10^-10^ | 51.9 (0.149) |
|  |  |  |  |  |  |  | Wald |  | 4.81x10^-24^ |  | 0.010 | 1.48x10^-21^ |  |
|  |  |  |  |  |  |  | Wald (interaction) |  | 1.02x10^-24^ |  | 0.006 | 4.99x10^-22^ |  |
| rs1424233 | *MAF* | 16 | 78240252 | T | C | 0.471 | SNP | -0.001 (0.002) | 0.553 | -0.001 (0.005) | 0.783 | 0.514 | 0 (0.975) |
|  |  |  |  |  |  |  | Wald |  | 0.843 |  | 0.091 | 0.730 |  |
|  |  |  |  |  |  |  | Wald (interaction) |  | 0.795 |  | 0.158 | 0.719 |  |
| rs571312 | *MC4R* | 18 | 55990749 | A | C | 0.234 | SNP | 0.014 (0.002) | 3.54x10^-8^ | 0.006 (0.006) | 0.361 | 4.94x10^-8^ | 34.9 (0.215) |
|  |  |  |  |  |  |  | Wald |  | 5.10x10^-11^ |  | 0.085 | 1.27x10^-9^ |  |
|  |  |  |  |  |  |  | Wald (interaction) |  | 1.75x10^-10^ |  | 0.059 | 2.81x10^-9^ |  |
| rs29941 | *KCTD15* | 19 | 39001372 | G | A | 0.679 | SNP | 0.004 (0.002) | 0.083 | 0.005 (0.005) | 0.361 | 0.051 | 0 (0.872) |
|  |  |  |  |  |  |  | Wald |  | 0.246 |  | 0.824 | 0.499 |  |
|  |  |  |  |  |  |  | Wald (interaction) |  | 0.237 |  | 0.742 | 0.446 |  |
| rs2075650 | *TOMM40* | 19 | 50087459 | A | G | 0.863 | SNP | 0.002 (0.003) | 0.472 | 0.004 (0.007) | 0.603 | 0.388 | 0 (0.84) |
|  |  |  |  |  |  |  | Wald |  | 0.766 |  | 0.751 | 0.874 |  |
|  |  |  |  |  |  |  | Wald (interaction) |  | 0.679 |  | 0.726 | 0.812 |  |
| rs2287019 | *QPCTL* | 19 | 50894012 | C | T | 0.813 | SNP | 0.002 (0.003) | 0.367 | -0.007 (0.007) | 0.274 | 0.673 | 45.3 (0.177) |
|  |  |  |  |  |  |  | Wald |  | 0.351 |  | 0.320 | 0.391 |  |
|  |  |  |  |  |  |  | Wald (interaction) |  | 0.581 |  | 0.239 | 0.558 |  |
| rs3810291 | *TMEM160* | 19 | 52260843 | A | G | 0.654 | SNP | 0.002 (0.003) | 0.338 | 0.008 (0.006) | 0.179 | 0.163 | 0 (0.379) |
|  |  |  |  |  |  |  | Wald |  | 0.004 |  | 0.937 | 0.063 |  |
|  |  |  |  |  |  |  | Wald (interaction) |  | 0.002 |  | 0.897 | 0.036 |  |

Chr., Chromosome; bp, basepair.

^a^Positions according to Build 36. ^b^The SNPs are aligned to the adult BMI increasing allele. ^c^Average effect allele frequency reported in the Metal meta-analysis of ALSPAC and Raine

Supplementary Table 6: For each loci reaching genome-wide significance (P<5x10^-8^), the results from the top reported SNP from the previously published studies are presented.

| Nearest Gene | Published SNP | LD (r^2^/D^’^) | Results from meta-analysis in childhood | | | | | |
| --- | --- | --- | --- | --- | --- | --- | --- | --- |
|  |  |  | A1 | A2 | Freq1 | Effect | Beta (SE) | P-Value |
| *ADCY3* | rs713586 (23) | 0.905/1.000 | C | T | 0.489 | SNP effect at age 8 | 0.011 (0.002) | P=2.39x10^-8^ |
|  |  |  |  |  |  | Global Wald | -- | 1.33x10^-8^ |
|  |  |  |  |  |  | Wald test for age interaction | -- | 0.293 |
| *OLFM4* | rs9568856 (18) | 0.690/0.906 | A | G | 0.127 | SNP effect at age 8 | 0.014 (0.003) | 1.68x10^-6^ |
|  |  |  |  |  |  | Global Wald | -- | 3.24x10^-6^ |
|  |  |  |  |  |  | Wald test for age interaction | -- | 0.002 |
| *FTO* | rs9939609 (24) | 0.934/0.966 | A | T | 0.392 | SNP effect at age 8 | 0.012 (0.002) | 1.09x10^-9^ |
|  |  |  |  |  |  | Global Wald | -- | 1.01x10^-20^ |
|  |  |  |  |  |  | Wald test for age interaction | -- | 3.72x10^-21^ |
| *MC4R* | rs571312  (23) (same SNP as reported in Table 2 | 1/1 | A | C | 0.227 | SNP effect at age 8 | 0.013 (0.002) | 4.94x10^-8^ |
|  |  |  |  |  |  | Global Wald | -- | 1.27x10^-9^ |
|  |  |  |  |  |  | Wald test for age interaction | -- | 2.81x10^-9^ |

**References:**

1. Howe LD, Parmar PG, Paternoster L, Warrington NM, Kemp JP, Briollais L, et al. Genetic influences on trajectories of systolic blood pressure across childhood and adolescence. Circulation Cardiovascular genetics. 2013;6(6):608-14.

2. Kemp JP, Medina-Gomez C, Estrada K, St Pourcain B, Heppe DH, Warrington NM, et al. Phenotypic dissection of bone mineral density reveals skeletal site specificity and facilitates the identification of novel Loci in the genetic regulation of bone mass attainment. PLoS Genet. 2014;10(6):e1004423.

3. Huang RC, Burke V, Newnham JP, Stanley FJ, Kendall GE, Landau LI, et al. Perinatal and childhood origins of cardiovascular disease. Int J Obes Res. 2006.

4. Scott MA, Norman RG, Berger KI. Modelling growth and decline in lung function in Duchenne's muscular dystrophy with an augmented linear mixed effects model. Journal of the Royal Statistical Society: Series C (Applied Statistics). 2004;53(3):507-21.

5. Warrington NM, Howe LD, Wu YY, Timpson NJ, Tilling K, Pennell CE, et al. Association of a Body Mass Index Genetic Risk Score with Growth throughout Childhood and Adolescence. PloS one. 2013;8(11):e79547.

6. Warrington NM, Wu YY, Pennell CE, Marsh JA, Beilin LJ, Palmer LJ, et al. Modelling BMI Trajectories in Children for Genetic Association Studies. PloS one. 2013;8(1):e53897.

7. Warrington NM, Tilling K, Howe LD, Paternoster L, Pennell CE, Wu YY, et al. Robustness of the linear mixed effects model to error distribution assumptions and the consequences for genome-wide association studies. Statistical applications in genetics and molecular biology. 2014.

8. Royall RM. Model Robust Confidence Intervals Using Maximum Likelihood Estimators. International Statistical Review / Revue Internationale de Statistique. 1986;54(2):221-6.

9. Speliotes EK, Willer CJ, Berndt SI, Monda KL, Thorleifsson G, Jackson AU, et al. Association analyses of 249,796 individuals reveal 18 new loci associated with body mass index. Nature genetics. 2010;42(11):937-48.

10. Yang J, Loos RJ, Powell JE, Medland SE, Speliotes EK, Chasman DI, et al. FTO genotype is associated with phenotypic variability of body mass index. Nature. 2012;490(7419):267-72.

11. Lango Allen H, Estrada K, Lettre G, Berndt SI, Weedon MN, Rivadeneira F, et al. Hundreds of variants clustered in genomic loci and biological pathways affect human height. Nature. 2010;467(7317):832-8.

12. Wood AR, Esko T, Yang J, Vedantam S, Pers TH, Gustafsson S, et al. Defining the role of common variation in the genomic and biological architecture of adult human height. Nat Genet. 2014;46(11):1173-86.

13. Heid IM, Jackson AU, Randall JC, Winkler TW, Qi L, Steinthorsdottir V, et al. Meta-analysis identifies 13 new loci associated with waist-hip ratio and reveals sexual dimorphism in the genetic basis of fat distribution. Nat Genet. 2010;42(11):949-60.

14. Berndt SI, Gustafsson S, Magi R, Ganna A, Wheeler E, Feitosa MF, et al. Genome-wide meta-analysis identifies 11 new loci for anthropometric traits and provides insights into genetic architecture. Nat Genet. 2013;45(5):501-12.

15. Dupuis J, Langenberg C, Prokopenko I, Saxena R, Soranzo N, Jackson AU, et al. New genetic loci implicated in fasting glucose homeostasis and their impact on type 2 diabetes risk. Nat Genet. 2010;42(2):105-16.

16. Morris AP, Voight BF, Teslovich TM, Ferreira T, Segre AV, Steinthorsdottir V, et al. Large-scale association analysis provides insights into the genetic architecture and pathophysiology of type 2 diabetes. Nat Genet. 2012;44(9):981-90.

17. Perry JR, Day F, Elks CE, Sulem P, Thompson DJ, Ferreira T, et al. Parent-of-origin-specific allelic associations among 106 genomic loci for age at menarche. Nature. 2014;514(7520):92-7.

18. Bradfield JP, Taal HR, Timpson NJ, Scherag A, Lecoeur C, Warrington NM, et al. A genome-wide association meta-analysis identifies new childhood obesity loci. Nat Genet. 2012;44(5):526-31.

19. Horikoshi M, Yaghootkar H, Mook-Kanamori DO, Sovio U, Taal HR, Hennig BJ, et al. New loci associated with birth weight identify genetic links between intrauterine growth and adult height and metabolism. Nat Genet. 2013;45(1):76-82.

20. van der Valk RJ, Kreiner-Moller E, Kooijman MN, Guxens M, Stergiakouli E, Saaf A, et al. A novel common variant in DCST2 is associated with length in early life and height in adulthood. Human molecular genetics. 2014.

21. Taal HR, St Pourcain B, Thiering E, Das S, Mook-Kanamori DO, Warrington NM, et al. Common variants at 12q15 and 12q24 are associated with infant head circumference. Nat Genet. 2012;44(5):532-8.

22. Cousminer DL, Berry DJ, Timpson NJ, Ang W, Thiering E, Byrne EM, et al. Genome-wide association and longitudinal analyses reveal genetic loci linking pubertal height growth, pubertal timing and childhood adiposity. Hum Mol Genet. 2013;22(13):2735-47.

23. Speliotes EK, Willer CJ, Berndt SI, Monda KL, Thorleifsson G, Jackson AU, et al. Association analyses of 249,796 individuals reveal 18 new loci associated with body mass index. Nat Genet. 2010;42(11):937-48.

24. Frayling TM, Timpson NJ, Weedon MN, Zeggini E, Freathy RM, Lindgren CM, et al. A common variant in the FTO gene is associated with body mass index and predisposes to childhood and adult obesity. Science (New York, NY). 2007;316(5826):889-94.
